# A simple and efficient strategy to produce transgene-free gene edited plants in one generation using paraquat resistant 1 as a selection marker

**DOI:** 10.3389/fpls.2022.1051991

**Published:** 2023-01-09

**Authors:** Xiangjiu Kong, Wenbo Pan, Tingyu Zhang, Lijing Liu, Huawei Zhang

**Affiliations:** ^1^ The Key Laboratory of Plant Development and Environmental Adaptation Biology, Ministry of Education, School of Life Sciences, Shandong University, Qingdao, China; ^2^ Peking University Institute of Advanced Agricultural Sciences, Weifang, China; ^3^ Shandong Laboratory of Advanced Agricultural Sciences, Weifang, China

**Keywords:** transgene-free gene edited plant, paraquat resistant 1, selection marker, CRISPR/Cas9, *Agrobacterium*-mediated transformation

## Abstract

**Introduction:**

DNA integration is a key factor limiting the marketing of CRISPR/Cas9-mediated gene edited crops. Several strategies have been established to obtain transgene-free gene edited plants; however, these strategies are usually time-consuming, technically difficult, providing low mutagenesis efficiency, and/or including a narrow host range.

**Method:**

To overcome such issues, we established a paraquat resistant 1 (PAR1)-based positive screening (PARS) strategy, which achieved efficient screening of transgene-free gene edited plants.

**Results:**

With PARS, the screening efficiency of mutant increased by 2.81-fold on average, and approximately 10% of T1 plants selected via PARS were transgenefree. Moreover, heritable transgene-free mutations at target loci were identified in the T1 generation.

**Discussion:**

Based on the previous reports and our data, we know that paraquat is toxic to all green plants, PAR1 is conserved among all plant species tested, and the transient expression of Cas9 editor can produce transgene-free gene edited plants. Thus, we assume that the PARS strategy established here has the potential to be widely used to screen transgene-free mutants in various crops using diverse CRISPR/Cas9 delivery approaches.

## Introduction

The clustered regularly interspaced short palindromic repeats (CRISPR)/CRISPR-associated endonuclease 9 (Cas9) system has been applied to multiple plant species for gene editing to facilitate studies on gene function and crop improvement (Chen et al., 2019; Gaillochet et al., 2021; Gao, 2021). However, the integration of the CRISPR/Cas9 construct can lead to phenotype instability, internal gene expression disturbance, and concerns related to genetically modified organism-associated legislation (Jones, 2015; Kim and Kim, 2016; Turnbull et al., 2021). Therefore, transgene-free gene edited plants is ideal for gene functional studies and agricultural applications. Two strategies are typically applied to produce transgene-free gene edited plants: (1) elimination of the integrated CRISPR/Cas9 construct *via* genetic segregation and (2) transient expression of the Cas9 editor (Gu et al., 2021). The first strategy has been used extensively. Moreover, it is suitable for most species that can be transformed with *Agrobacterium*. However, this strategy requires the selection of transgene-free gene edited plants from the progenies of transgenic plants, which is laborious and unfeasible in vegetatively propagated plants, such as potato or trees, with a long juvenile period (Gao et al., 2016; Lu et al., 2017; He et al., 2018; He et al., 2019; Stuttmann et al., 2021). The second strategy delivers DNA, *in vitro*-transcribed RNA, or preassembled CRISPR/Cas9 ribonucleoproteins to protoplasts, zygotes, and embryo cells *via* particle bombardment or polyethylene glycol Ca^2+^ (Svitashev et al., 2016; Zhang et al., 2016a; Liang et al., 2017; Park and Choe, 2019). These approaches are mostly technically difficult and inefficient. Therefore, an inexpensive, convenient, and highly efficient approach is required for producing transgene-free gene edited plants.

*Agrobacterium*-mediated transformation is a low-cost and simple method to deliver Cas9 editors in plant cells (Hwang et al., 2017). More importantly, transgene-free gene edited plants have been detected in regenerated seedlings from cells inoculated with *Agrobacterium* carrying the CRISPR/Cas9 construct without selection (Chen et al., 2018). However, the efficiency of this strategy is relatively low, as most of the regenerated seedlings are unmutated. Thus, a screening marker is required to enrich the transgene-free gene edited plants. Previous studies have shown that some exogenous and endogenous genes can be used as co-editing markers to improve screening efficiency. For example, the hygromycin resistance gene (HygR) in construct and GLABRA2 (*GL2*) in genome (Xu et al., 2020; Kong et al., 2021). Whereas, these markers are not conducive to the screening of transgene-free gene edited plants. We assume some genes that provide plants with herbicide or antibiotic resistance when mutated, e.g., acetolactate synthase (*ALS*) and multiple antibiotic resistance 1 (*MAR1*), can be used as markers for this purpose (Aufsatz et al., 2009; Zhang et al., 2019; Rinne et al., 2021). However, *ALS* can only serve as a selection marker for base editors because some point mutation forms of *ALS*, rather than its knockouts, provide herbicide resistance to plants (Yu and Powles, 2014). Null mutation of *MAR1* results in plant resistance to several aminoglycoside antibiotics, including kanamycin, streptomycin, gentamicin, etc. (Rinne et al., 2021). Among these antibiotics, kanamycin has been applied to select transgenic plants in multiple plants but works inefficiently in some species, such as tomato (Honda et al., 2021). Paraquat resistant 1 (*PAR1*), a gene that encodes a putative L-type amino acid transporter protein localized to the Golgi apparatus, was screened from an *Arabidopsis* ethyl methanesulfonate (EMS) mutant library, and its paraquat resistance phenotype was further confirmed by analyzing its T-DNA mutants, indicating that the *par1* loss-of-function mutant could be selected through paraquat treatment. Importantly, paraquat is nonselective herbicide for green plants (Nazish et al., 2022) and *par1* mutants exhibit no obvious developmental defects in *Arabidopsis* (Li et al., 2013). However, it is not clear whether *PAR1* can be used as a screening marker for CRISPR/Cas9.

Here, we created *par1* mutants in *Arabidopsis* using the CRISPR/Cas9 system, which revealed that abolishing the function of PAR1 exhibited a strong paraquat-resistant phenotype without growth penalties under both normal growth and several stress conditions. We also confirmed that *PAR1* can be used as a coediting marker to enrich mutants of target loci. Furthermore, we found that transgene-free gene edited plants could be easily detected in the T1 generation using *PAR1* as a selection marker. Given that PAR1 is a conserved protein in all plant species tested, we proposed that this *PAR1*-based positive screening (hereafter, referred to as PARS) strategy may be applicable for various plant species and multiple CRISPR/Cas9 delivery approaches.

## Materials and methods

### Plant growth conditions, transformation, and selection

*Arabidopsis thaliana* accession Col-0 was used as the wild type. Seeds were surface sterilized and plated on 1/2 Murashige and Skoog (MS) medium containing 2.2% (w/v) MS basal salts, 1.5% (w/v) sucrose, 0.05% (w/v) MES (pH 5.7) and 0.8% (w/v) agar or in soil with a 16 h light/8 h dark photoperiod at 22 °C. *Agrobacterium*-mediated transformation of plants was performed by floral dip (Clough and Bent, 1998). To select transgenic plants, T0 seeds were surface sterilized and sown on 1/2 MS medium containing 25 mg/L hygromycin. While for transgene-free gene edited plants selection, T0 seeds were sown on 1/2 MS medium containing 1 μM paraquat. After 14 days of growth on sterile agar plates, resistant seedlings were transferred to soil. To further confirm the T1 edited plants without Cas9, their seeds were sown on 1/2 MS medium supplemented with 25 mg/L hygromycin for two weeks

### Construction of CRISPR/Cas9 vectors

Four single guide RNAs (sgRNAs), i.e., sgRNA*_par1_* 1–4, targeting *PAR1* were designed using the online predictor tool CCTop (https://cctop.cos.uni-heidelberg.de/), and the efficacy score and mismatch positions were used as evaluation criteria. Additionally, sgRNAs targeting jasmonate-ZIM-domain protein 1 (*JAZ1*) and gibberellic acid insensitive (*GAI*) were used as previously described (Kong et al., 2021). Each oligonucleotide pair coding for the designed sgRNAs was annealed to form double-stranded DNA (dsDNA). The sequences of the synthesized DNA oligonucleotides and all primers used in this study are listed in **Supplementary Table S1**.

To construct pHEE401E (Wang et al., 2015) vectors targeting four different sites of *PAR1* and one site of *JAZ1* or *GAI*, the dsDNA was fused to the Bs*a*I-digested vector pHEE401E using T4 DNA ligase (EL0011, Thermo Scientific™). To construct a new vector named pPARS, which targets *PAR1* as a selection marker, the DNA sequence of sgRNA*_par1_* 3 was introduced into the *PAR1*-2Tar-R primer when the primer was synthesized. The primer pair PAR1-2Tar-F/R (**Supplementary Table S1**) was then used to amplify a sequence containing the U6 terminator-U6 promoter-sgRNA*_par1_* 3 element using the pCBC-DT1T2 plasmid as a template (Xing et al., 2014). Through homologous recombination using ClonExpress^®^ II One Step Cloning Kit (C112, Vazyme Biotech co., ltd.), this element was ligated into the pHEE401E binary vector digested by *Bsa*I. Additionally, a *Bsa*I restriction site was added to the PAR1-2Tar-F/R primers; hence, the final vector retained the *Bsa*I cleavage site, allowing the insertion of sgRNAs for the target loci of interest. DNA sequence for pPARS plasmid was shown in **Supplementary Data Set S1**. To produce the pPARS constructs targeting *JAZ1* or *GAI*, their dsDNAs were introduced into the *Bsa*I sites of pPARS vectors. *Agrobacterium tumefaciens* strain GV3101 was transformed with the final binary vectors for floral dip.

### Genotyping of the transgenic plants

Genomic DNA was extracted from 4-week-old plant leaves using DNA extraction buffer [50 mM Tris–HCl (pH 7.5), 300 mM NaCl, and 300 mM sucrose]. To confirm the integration of T-DNA or CRISPR/Cas9-introduced mutations of the target gene, gene-specific primers (**Supplementary Table S1**) were used for PCR amplifications with *EasyTaq* ^®^ DNA Polymerase (AP111; TransGen Biotech Co., Ltd.) under the following cycling conditions: initial denaturation at 95°C for 5 min; 35 cycles at 95°C for 30 s, 58°C for 30 s, and 72°C for 60 s; and final extension at 72°C for 5 min. The PCR products were visualized on a 1% agarose gel, and samples with no Cas9-specific band were considered transgene-free gene edited plants. Additionally, Sanger sequencing was performed to identify mutations in the target region. All sequencing data were analyzed using online software (http://shinyapps.datacurators.nl/tide/ and https://ice.synthego.com/#/).

### Biomass and flowering time analysis

To detect the fresh weight of Col-0 and *par1*, plants were sown in soil and grew in chamber with a 12 h light/12 h dark photoperiod at 22°C. After three and a half weeks, plant weight was determined. For the determination of flowering time, Col-0 and *par1* were grown in soil at 22°C under 16-h light/8-h dark cycles. Flowering time was estimated by counting the number of rosette leaves according to the visible flower buds at the center of the rosette and the days from sowing to flowering.

### Root growth assay

To analyze whether *PAR1* affects plants to cope with multiple stresses, WT and *par1* were sown on 1/2 MS medium vertically with or without 200 mM mannitol, 150 mM NaCl or 8 mM NaHCO_3_, respectively, and growing under 12 h light/12 h dark photoperiod at 22°C for 12 d. To detect the response of WT and *par1* to low temperature, WT and *par1* were sown on 1/2 MS medium vertically and growing under 12 h light/12 h dark photoperiod at 12°C for 12 d. To evaluate whether *PAR1* mutation affects the functional analysis of target genes, *Arabidopsis* seeds of WT, T1 seeds of *par1 #*10 and *jaz1 par1 #*17 were surface sterilized and sown on 1/2 MS medium vertically with or without 50 μM methyl-jasmonate (JA) for 10 d, following which primary root length was measured using the ImageJ software (https://imagej.nih.gov/).

### Sequence alignment and phylogenetic analyses

Amino acid sequences of PAR1 in different species were obtained from NCBI (http://www.ncbi.nlm.nih.gov). Multiple sequence alignment was performed using the ClustalW algorithm with 1,000 bootstrapping trials and the phylogenetic analysis was conducted using the neighbor-joining method in MEGA 7.0.14 software with 1000 bootstrap value.

### Statistical analysis

Statistical analyses were performed using Graph Pad Prism 8.0. The statistical significance levels (value of p<0.05) was determined by Student’s t-test or one-way ANOVA.

### Accession numbers

*PAR1*: *At1g31830*; *JAZ1*: *At1g19180*; *GAI*: *At1g14920*.

## Results

### Design of the PARS strategy

**Figure 1:**
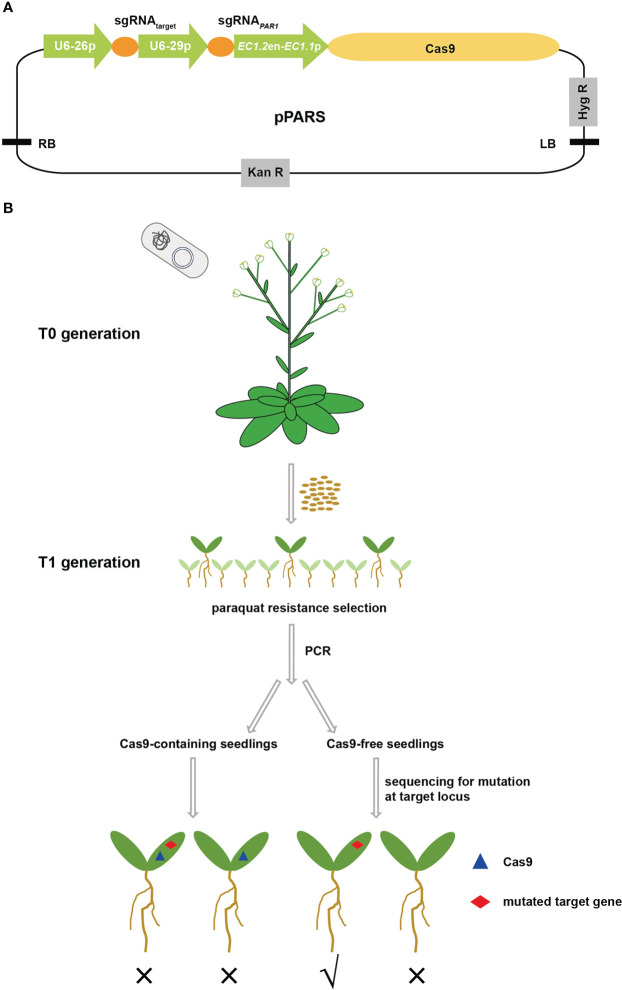
Schematic showing the PARS strategy to screen transgene-free gene edited plants. **(A)** The structure of the pPARS plasmid. The expression of sgRNA*_PAR1_* and sgRNA*_target_* is driven by the U6-29 and U6-26 promoters, respectively. The Cas9 expression cassette includes the *EC1* promoter (*EC1.2* enhancer plus *EC1.1* promoter) and the CDS of *Cas9*. Hyg R, hygromycin resistance gene; Kan R, kanamycin resistance gene; RB and LB, T-DNA right and left borders, respectively. **(B)** Outline of the PARS strategy. pPARS constructed with sgRNA_target_ was transformed into Col-0 plants by floral dip. T0 seeds were collected and sown on paraquat-containing plates to select paraquat-resistant plants, which are shown as large green seedlings. The DNA of paraquat-resistant T1 seedlings was extracted, and PCR was performed using Cas9-specific primers to select transgene-free plants. The Cas9-containing seedlings were discarded, and Cas9-free seedlings were further sequenced for mutation at the target locus to obtain transgene-free gene edited plants. The blue triangle on the seedling indicates insertion of Cas9, and the red diamond indicates mutation at the target site. Portions of the images were modified from the Microsoft PowerPoint clip art database.

As a screening marker of the CRISPR/Cas9 system, its mutants should be easily screened out under certain conditions and should not have obvious fitness costs for subsequent application. Using a literature search, we confirmed that *PAR1* met these necessary criteria. PAR1 is a Golgi-localized transporter that may be responsible for the uptake of paraquat into chloroplasts to generate toxic reactive oxygen species (Li et al., 2013). Knockout of *PAR1* resulted in a paraquat-resistant phenotype and moreover showed no growth defect, which perfectly fit our requirements. Thus, we designed the PARS screening strategy to efficiently select transgene-free gene edited plants based on *PAR1* mutation. First, the expression cassette of *PAR1* sgRNA driven by the *AtU6* promoter was cloned into pHEE401E to construct the pPARS vector (**Figure 1A**), which has an additional sgRNA expression cassette for the target site and a Cas9 expression cassette driven by the enhancer of egg-cell specific promoter *EC1.2* fused to the *EC1.1* promoter, thus reducing the ratio of chimeric mutants (Wang et al., 2015). The sgRNA for the target locus was then introduced into pPARS, and the final construct was used to transform *Arabidopsis* plants *via* floral dip (**Figure 1B**). The seeds of T0 plants were collected and sown on 1/2 MS plates supplemented with paraquat to screen *par1* mutants. Additionally, DNA was extracted from the *par1* mutants to determine whether they were transgene-free mutants *via* PCR with Cas9 primers. Finally, the transgene-free T1 plants were sequenced to determine whether mutations occurred at target loci (**Figure 1B**).

### *PAR1* is a suitable marker for the selection of CRISPR/Cas9-created mutants

**Figure 2:**
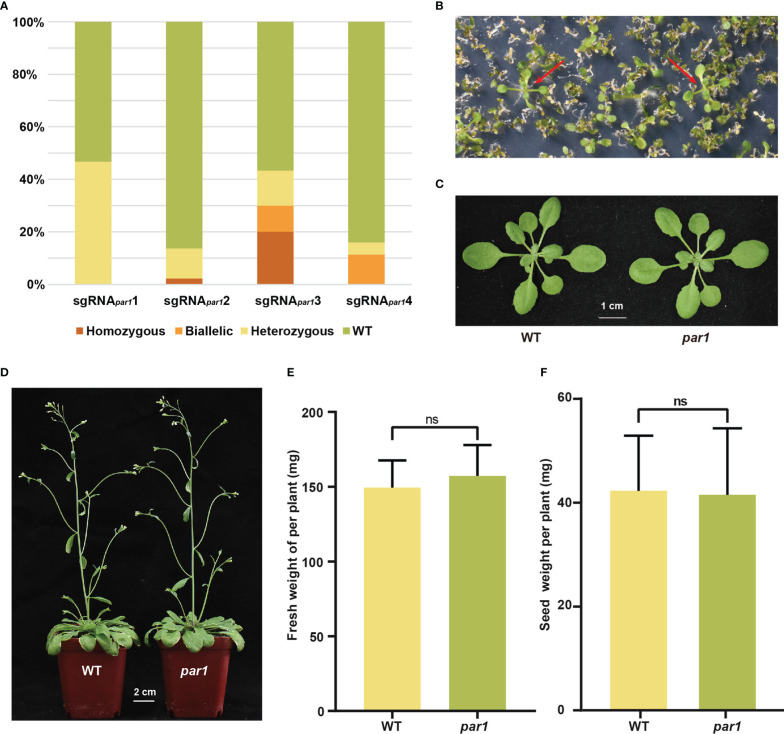
*PAR1* is a suitable selection marker for transgene-free gene edited plants. **(A)** Percentages of WT and different types of mutations at four sgRNA*_PAR1_* target loci among their T1 plants. **(B)** Selection of the *par1* mutant on 1/2 MS medium supplemented with 1 μM paraquat. The large and green seedlings indicated by red arrows are paraquat-resistant seedlings. **(C)** Representative three-week-old WT and *par1* mutant plants. **(D)** Representative six-week-old WT and *par1* mutant plants. **(E)** The fresh weight per plant of WT and *par1* mutant (*n* = 20) growing in soil with a 12 h light/12 h dark photoperiod at 22°C for three and a half weeks. **(F)** Seed weight per plant for WT and the *par1* mutant (*n* = 30). Data are shown as mean ± SD. ns, no significant difference.

To select a suitable sgRNA for *PAR1*, we designed four sgRNAs for this gene. As shown in **Supplementary Figure S1**, sgRNA*_par1_* 1–4 targeted 79, 118, 313, and 905 bp downstream of the translation start site ATG. We obtained ≥ 30 T1 plants for each sgRNA*_par1,_* and all T1 plants of the four sgRNA*_par1_* s were sequenced. The mutation rates of the four target sites were 46.67%, 13.64%, 43.33%, and 15.91%, respectively, and sgRNA*_par1_* 3 exhibited the highest homozygous and biallelic mutation efficiency, i.e., 20% (6/30) and 10% (3/30) of the T1 plants were homozygous and biallelic, respectively (**Figure 2A** and **Supplementary Table S2**). Therefore, sgRNA*_par1_* 3 was used to construct pPARS. A homozygous *par1* mutant created using sgRNA*_par1_* 3 was selected to determine whether the *par1* mutant can be easily screening out using paraquat and has no growth penalty as reported (Li et al., 2013). To determine the feasibility of screening *par1* mutants using paraquat, we mixed 25 *par1* homozygous seeds with 0.2 g of WT seeds and selected them using 1 μM paraquat according to a previously published method (Li et al., 2013) All plants screened using 1 μM paraquat were *par1* mutants (**Figure 2B** and **Supplementary Figure S2**), indicating the screening with 1 μM paraquat is very efficient. To determine whether the *par1* mutants exhibit growth defects, we compared the growth statuses of three- and six-week-old WT and *par1* mutant plants. As shown in **Figures 2C, D**, the growth of the *par1* mutant and WT did not differ significantly at these stages. We also measured the biomass, flowering time, and seed weight per plant of the WT and *par1* mutant and found no significant difference between them (**Figures 2E, F** and **Supplementary Figure S3**). Importantly, mutating *PAR1* exerted no negative effects on plant growth under multiple stress conditions (**Supplementary Figure S3**). Collectively, these results suggest that *PAR1* is a suitable marker for selecting gene edited CRISPR/Cas9-created mutants.

### Transgene-free *PAR1*-edited plants can be screened from T1 plants

**Figure 3:**
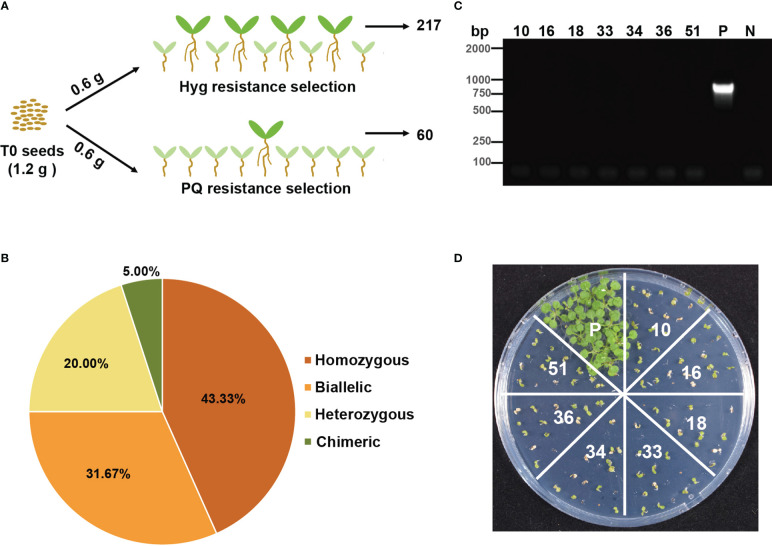
Transgene-free *par1* mutants could be detected when T0 seeds were selected by paraquat. **(A)** Outline of selecting equal amount of T0 seeds transformed with sgRNA*par1* by hygromycin and paraquat. **(B)** Percentages of different types of mutations at the *PAR1* locus among all T1 plants selected on paraquat plates. **(C)** The PCR results of transgene-free T1 plants amplified with *Cas*9-specific primers. P: positive control (PCR with DNA extracted from a transgenic plant containing Cas9). N: no sample control (PCR with no DNA sample). **(D)** The progenies of transgene-free *par1* mutants are sensitive to hygromycin. Seeds of these transgene-free T1 plants were sown on 1/2 MS plates supplemented with 25 mg/L hygromycin for two weeks.

As we know, transiently expressing Cas9 and sgRNA can create transgene-free gene edited plants (Zhang et al., 2016a), and furthermore, transgene-free gene edited plants were obtained in plants that were regenerated from calli transiently expressing Cas9 from T-DNA delivered by *Agrobacterium* without selection (Chen et al., 2018). We assumed that the transgene-free *par1* mutant could be detected in T1 plants if we selected T0 seeds by paraquat. For this purpose, we equally divided T0 seeds (1.2 g) obtained from the same transformation event (with sgRNA*_par1_* 3) into two groups, sowing these seeds on 1/2 MS plates supplemented with either hygromycin or paraquat. In total, 217 and 60 positive seedlings were observed on hygromycin and paraquat plates, respectively (**Figure 3A**). We sequenced all positive seedlings and found that 63 of 217 hygromycin-resistant plants were *par1* mutants, with 26.98% (17), 12.70% (8), 53.97% (34), and 6.35% (4) being homozygous, biallelic, heterozygous, and chimeric, respectively (**Supplementary Figure S4** and **Supplementary Table S2**). All 60 paraquat-resistant seedlings were *par1* mutants, with 43.33% homozygotes (26), 31.67% biallelic mutants (19), 20.00% heterozygotes (12), and 5.00% chimeras (3) (**Figure 3B** and **Supplementary Table S2**). We were surprised to detect heterozygotes in the paraquat plates because the *par1* mutation is recessive according to a previous report (Li et al., 2013). However, the ratio of homozygotes and biallelic mutants was higher and that of heterozygotes was lower in paraquat-resistant plants than in hygromycin-resistant *par1* mutants, indicating that the screening is efficient. Given that increasing the concentration of paraquat in selection medium is lethal to >20% of *par1* homozygotes, we continued to use 1 μM paraquat for the following experiments (Li et al., 2013).

The above results indicate that more *par1* homozygous and biallelic mutants were obtained from paraquat plates than from hygromycin plates with the same amount of T0 seeds; therefore, some *par1* mutants selected on paraquat plates were likely transgene-free. To confirm this hypothesis, Cas9-specific primers were used to validate the integration of T-DNA in paraquat-selected plants. We found that 11.67% (7/60) of paraquat-resistant plants were transgene-free (**Figure 3C** and **Supplementary Figure S5A**), and all of them were *par1* homozygous or biallelic mutants (**Supplementary Figure S5B**). Subsequently, we sowed the T1 seeds of these plants on hygromycin plates and found that they were all hygromycin-sensitive, supporting the finding that these lines were transgene-free (**Figure 3D**). Overall, these results indicate that *PAR1* can be used as a screening marker to obtain transgene-free gene edited plants using the CRISPR/Cas9 system.

### PARS strategy enhances screening efficiency at target sites

**Figure 4:**
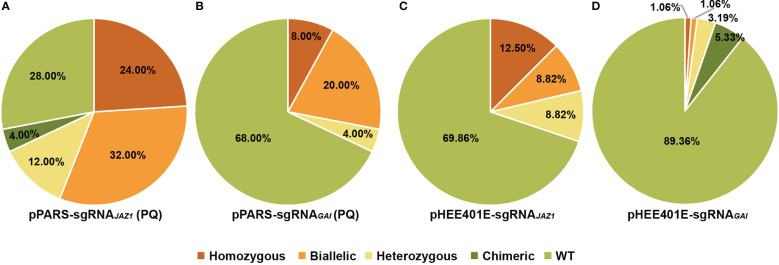
Mutation type and frequency at target sites with or without PARS. **(A)** Percentages of WT and different types of mutations at *JAZ1* loci selected using PARS on paraquat (PQ) plates. **(B)** Percentages of WT and different types of mutations at *GAI* loci selected using PARS on paraquat (PQ) plates. **(C)** Percentages of WT and different types of mutations at *JAZ1* loci using the original system (pHEE401E). **(D)** Percentages of WT and different types of mutations at *GAI* loci using the original system (pHEE401E).

As co-editing is a common phenomenon in multiplex gene edited plants, adding selection markers has been shown to enrich the mutants of target sites created using the CRISPR/Cas9 system (Zhang et al., 2016b; Wang et al., 2019; Kong et al., 2021). We assumed that the *PAR1* locus could also serve as a selection marker to enhance the screening efficiency. Therefore, we constructed two sgRNAs targeting *JAZ1* and *GAI* on pPARS, and the final constructs were transformed into *Arabidopsis*. A third of the T0 seeds were selected on 1/2 MS supplemented with hygromycin, whereas two-thirds of these seeds were selected on paraquat plates. In total, 57 hygromycin-positive seedlings and 25 paraquat-positive seedlings were detected for *JAZ1*, and all seedlings were sequenced to identify the mutation form at the target. We found that the screening efficiency increased from 12.28% to 72.00% (**Figure 4A**, **Supplementary Figure S6A** and **Supplementary Table S2**). Furthermore, 56.00% of paraquat-positive plants were homozygous or biallelic *jaz1* mutants compared with only 5.27% of hygromycin-positive plants (**Figure 4A**, **Supplementary Figure S6A** and **Supplementary Table S2**). For the *GAI* target, we identified 70 hygromycin-positive seedlings with 12 *gai* mutants (17.14%) and 25 paraquat-positive seedlings with 8 *gai* mutants (32.00%) (**Figure 4B**, **Supplementary Figure S6B** and **Supplementary Table S2**). Among the selected seedlings, 4.29% (3) and 28.00% (7) were homozygous or biallelic mutants in hygromycin-positive and paraquat-positive seedlings, respectively (**Figure 4B**, **Supplementary Figure S6B** and **Supplementary Table S2**). Taken together, these findings confirm that PARS facilitates the mutant screening at target loci.

To rule out the effect of expressing two sgRNAs on editing efficiency, we constructed sgRNAs of *JAZ1* and *GAI* to pHEE401E and found that the mutation frequencies were 30.14% and 10.64%, respectively (**Figures 4C, D**). The homozygous/biallelic ratio of T1 seedlings was 21.32% for *JAZ1* and 2.12% for *GAI* (**Figures 4C, D** and **Supplementary Table S2**). Even though the mutation efficiency was affected by coexpressing sgRNA*_par1_* 3, PARS still facilitates mutant screening at target loci, especially the screening of homozygous and biallelic mutants. Based on our results, PARS increased screening efficiency by 2.03-fold for *JAZ1* and 3.58-fold for *GAI* compared with the original vector pHEE401E (**Figure 4** and **Supplementary Table S2**).

### Transgene-free gene edited plants with modifications at target sites can be obtained using PARS

**Figure 5:**
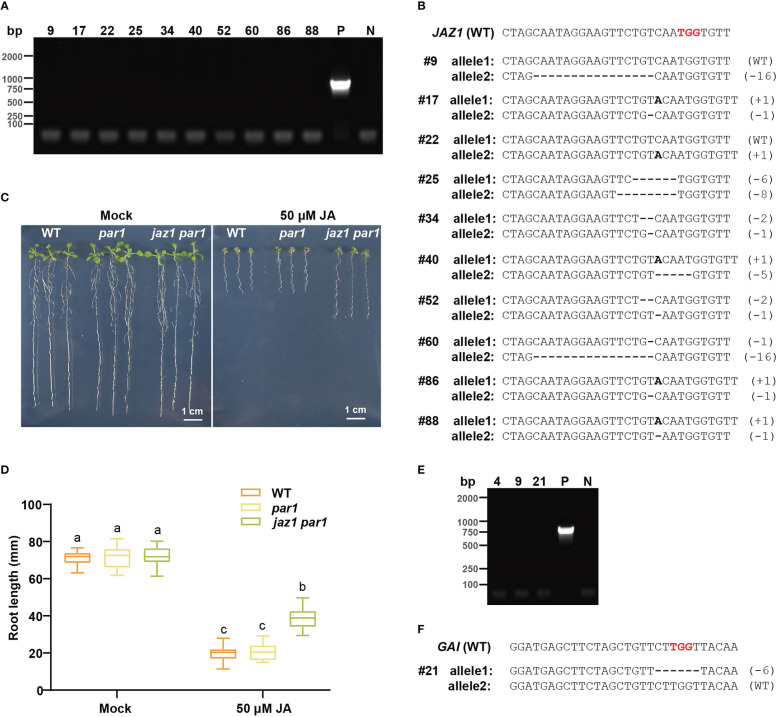
Transgene-free gene edited plants could be created by PARS strategy. **(A)** The PCR results of transgene-free T1 plants of pPARS-sgRNA*_JAZ1_* amplified with *Cas*9-specific primers. P: positive control (PCR with DNA extracted from a transgenic plant containing Cas9). N: no sample control (PCR with no DNA sample). **(B)** The DNA sequences of the transgene-free *jaz1* mutants. The reference sequence of WT is shown on the top. The PAM sequence is shown in red. These indels in mutant alleles are depicted by a dash (deletion) or bold letter (insertion). **(C)** Representative seedlings of WT, *par1* mutant and *jaz1 par1* double mutant grown on 1/2 MS plates with or without 50 μM JA. **(D)** The analysis of primary root length of WT, *par1* mutant and *jaz1 par1* double mutant grown on 1/2 MS plates with or without 50 μM JA. **(E)** The PCR results of transgene-free pPARS-sgRNA*_GAI_* T1 plants amplified with *Cas*9-specific primers. P: positive control (PCR with DNA extracted from a transgenic plant containing Cas9). N: no sample control (PCR with no DNA sample). **(F)** The DNA sequences of the transgene-free *gai* mutant. The reference sequence of WT is shown on the top. The PAM sequence is shown in red. The deletions in mutant allele are depicted by dashes.

To determine whether transgene-free plants with a modification at the target locus can be obtained using PARS, we retransformed the construct of pPARS-sgRNA*_JAZ1_* into 24 of *Arabidopsis* plants to obtain more T0 seeds, after which we selected all T0 seeds on 1/2 MS plates supplemented with 1 μM paraquat. In total, 106 paraquat-resistant seedlings were obtained for sgRNA*_JAZ1_*, 10 of which were transgene-free, as identified *via* PCR with Cas9-specific primers (**Figure 5A** and **Supplementary Figure S7A**). We sequenced these 10 transgene-free seedlings and found 8 biallelic mutants and 2 heterozygotes for the *JAZ1* locus (**Figure 5B**). For example, *#*17, a biallelic mutant for *JAZ1*, had a 1 bp insertion in one allele and a 1 bp deletion in the other allele (**Figure 5B**). The T1 seeds of all lines were hypersensitive to hygromycin, confirming that they were transgene-free mutants (**Supplementary Figure S7B**). JAZ1 belongs to a member of the transcriptional repressor family involved in jasmonic acid (JA) signaling, and the *JAZ1* sgRNA, designed to target the coding region of the JA-associated domain, generates mutants with compromised sensitivity to exogenous JA (Thines et al., 2007). Therefore, we sought to determine whether the *PAR1* mutation impedes the functional study of JAZ1. Accordingly, we sowed WT, *par1*, and *jaz1 par1* seeds on 1/2 MS plates supplemented with 50 μM MeJA, and observed no significant difference between the WT and *par1* mutant indicating PAR1 mutation does not change plant response to JA (**Figure 5C, D**). The *jaz1* mutant phenotype was further investigated in the *par1* background. Consistent with the results of a previous study (Kong et al., 2021), JA resistance was increased in the T2 seedlings of *jaz1 #*17 compared with that in control plants (**Figures 5C, D**). Collectively, these results indicate that the *PAR1* mutation does not affect the functional study of target genes.

We also showed that transgene-free *gai* mutant could be identified *via* PARS using pPARS-sgRNA*_GAI_*. Among all 33 paraquat-positive T1 plants for *GAI*, three transgene-free plants were detected (**Figure 5E** and **Supplementary Figure S8A**). Additionally, one transgene-free *plant* (line 21) was identified as *gai* heterozygous (**Figure 5F**). Furthermore, its progenies were hygromycin-sensitive (**Supplementary Figure S8B**). We believe that screening more T0 seeds on paraquat plates would lead to the identification of homozygous/biallelic mutants for *GAI*. These results support our claim that PARS is an appropriate strategy for obtaining transgene-free gene edited plants *via* the CRISPR/Cas9 system. ALS has been used as selection marker to obtain the transgene-free base edited plants (Zhang et al., 2019). As CRISPR-STOP could result in gene silence through base editor-created null mutations (Kuscu et al., 2017), we assume transgene-free base edited mutants could also be screened using PARS with PAR1 sgRNAs to generate stop codons.

### PARS has the potential to be applicable to multiple crop species

**Figure 6:**
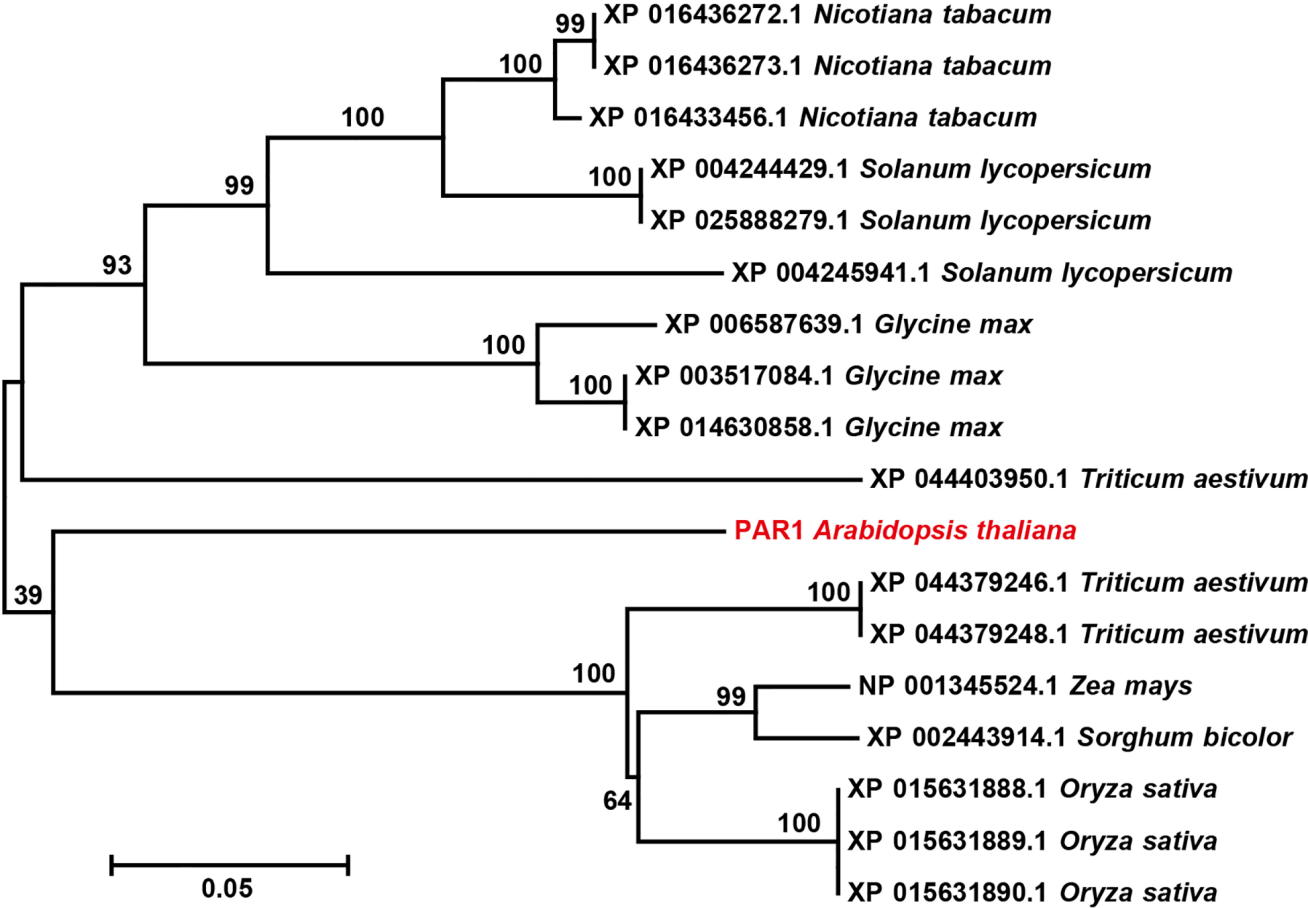
The phylogenetic analysis of PAR1 and PAR1 homologs in several crops. *Arabidopsis* PAR1is highlighted in red. The numbers above the branches indicate bootstrap values. The bar indicates a genetic distance of 0.05.

To determine whether the PARS strategy has the potential to be used for obtaining transgene-free gene edited crop plants, we investigated the conservation of PAR1 in different crop species. The *Arabidopsis* PAR1 amino acid sequence showed high similarity with its homologous proteins from *Nicotiana tabacum*, *Solanum lycopersicum*, *Glycine max*, *Triticum aestivum*, *Zea mays*, *Sorghum bicolor*, and *Oryza sativa* (**Supplementary Figure S9**). To further investigate the relationship between the PAR1 protein and its homologs in different species, a phylogenetic tree was generated with the neighbor - joining likelihood method using MEGA 7.0.14, which further confirmed PAR1 is a conserved protein (**Figure 6**). Since multiple PAR1 homologs were identified in each crop, e.g., three *PAR1*-like genes were observed in tomato, the *PAR1* homolog that drives the transport of paraquat in the corresponding plant must be identified to enable the selection of transgene-free gene edited plants using PARS. Additionally, whether eliminating the function of PAR1 homologs will harms crop development also needs to be considered before applying PARS in the corresponding crop.

## Discussion

To avoid the insertion of foreign DNA, multiple strategies have been established to transiently express genome editors in plants (Zhang et al., 2016a; Liang et al., 2017; Chen et al., 2018). However, most of these strategies are generally accompanied by low mutant frequency, as no selection marker can be used to exclude the unmodified seedlings. Thus, there is an urgent need for a method to easily screen gene edited plants out from all regenerated ones. The PARS strategy we developed in this study meets this need well. We believe *paraquat resistant 1* (*PAR1*) is a perfect selection marker to screen transgene-free gene edited plants based on the following two reasons. First, the loss-of-function mutant of *PAR1* could be easily screened out through paraquat treatment and no obvious developmental defects was detected in this study (**Figure 2**). Second, paraquat is a nonselective herbicide for green plants, and the *PAR1* gene is conserved in different plants, suggesting that this strategy has the potential to be adopted to other crops (**Figure 6**) (Nazish et al., 2022). We used *Agrobacterium* transformation, the most widely used approach of CRISPR/Cas9 delivery for plants, as an example to verify our hypothesis in this study. We believe PARS is also applicable to gene editing achieved with other transformation methods, such as particle bombardment. nanomaterials scenario and RNA- or RNP-mediated gene editing.

This study revealed that *PAR1* can serve as a selection marker to enrich screening efficiency at target loci (**Figure 4**). Because coediting frequently occurs when multiple sgRNAs are delivered in the same plant cells (Zhang et al., 2016b; Wang et al., 2019), multiple coselection strategies have been established to enrich edited plants at target loci (Zhang et al., 2019; Xu et al., 2020; Kong et al., 2021). For example, base editing on P174 of wheat ALS enriches the mutation frequency of the target by several fold (Zhang et al., 2019), and GLABRA2 mutation-based visible selection increased the screening efficiency by 2.58- to 7.50-fold in *Arabidopsis* (Kong et al., 2021). In the PARS system established here, the screening efficiency was increased on average by 2.81-fold (**Figure 4**). Moreover, 28% of *gai* mutants and 56% of *jaz1* mutants among T1 plants were homozygous/biallelic (**Figure 4**). Importantly, mutating *PAR1* had no obvious effect on the functional study of the *jaz1* mutant (**Figure 5**). We assume that transgene-free gene editing events mediated by Cas9, such as fragment deletion and insertion and fragment replacement, could also be enriched and screened using PARS. Moreover, PARS could also facilitate isolating transgene-free gene editing events mediated by base editor thorough creating stop codons in *PAR1* (Kuscu et al., 2017).

Paraquat, the third most commonly used herbicide in the world, is nonselective and has a broad spectrum (Nazish et al., 2022). Other than the *PAR1* mutation, paraquat tolerance 15-D (*PQT15-D*) is the dominant mutation that confers high resistance to paraquat (Xia et al., 2021). The overexpression of paraquat resistance genes, such as *Ochrobactrum anthropi pqrA* and *EiKCS*, also confers paraquat resistance in plants (Jo et al., 2004; Luo et al., 2022). Thus, paraquat could serve as a potential alternative for selecting transgenic plants, especially when the target plants are tolerant to conventional antibiotics such as kanamycin and hygromycin. To the best of our knowledge, no transgene-free paraquat-resistant crops have been developed to date owing to the lack of knowledge on the mechanisms underlying paraquat resistance (Nazish et al., 2022). If the functional PAR1 and PQT15 homologs that transport paraquat were identified in different crops, PAR1 and PQT15 would likely be the best editing targets for producing paraquat-resistant crops. The transgene-free gene edited plants obtained through the PARS strategy will also offer the possibility of crops with herbicide resistance, which is a major requirement in modern agriculture (Zhu et al., 2020).

The application of PARS should be extended to other gene editing methods, including zinc-finger nucleases (ZFNs) and transcription activator-like effector nucleases (TALENs) (Zhang et al., 2017; Shamshirgaran et al., 2022). As PAR1 is conserved among different plants tested (**Figure 6**), we propose that the PARS system has the potential to be widely used in the gene editing of various plant species using multiple gene editing systems.

## Data availability statement

The original contributions presented in the study are included in the article/**Supplementary Material**. Further inquiries can be directed to the corresponding author.

## Author contributions

HZ and LL conceived this research. XK performed all the experiments with the help of WP and TZ. LL and XK wrote the draft. All authors read and approved the final version.

## References

[B1] Aufsatz W. Nehlin L. Voronin V. Schmidt A. Matzke A. J. Matzke M. (2009). A novel strategy for obtaining kanamycin resistance in *Arabidopsis thaliana* by silencing an endogenous gene encoding a putative chloroplast transporter. Biotechnol. J. 4 (2), 224–229. doi: 10.1002/biot.200800156 19226556

[B2] Chen L. Li W. Katin-Grazzini L. Ding J. Gu X. Li Y.. (2018). A method for the production and expedient screening of CRISPR/Cas9-mediated non-transgenic mutant plants. Hortic. Res. 5, 13. doi: 10.1038/s41438-018-0023-4 29531752 PMC5834642

[B3] Chen K. Wang Y. Zhang R. Zhang H. Gao C. (2019). CRISPR/Cas genome editing and precision plant breeding in agriculture. Annu. Rev. Plant Biol. 70, 667–697. doi: 10.1146/annurev-arplant-050718-100049 30835493

[B4] Clough S. Bent A. (1998). Floral dip: A simplified method for agrobacterium-mediated transformation of *Arabidopsis thaliana* . Plant J. 16 (6), 735–743. doi: 10.1046/j.1365-313x.1998.00343.x 10069079

[B5] Gaillochet C. Develtere W. Jacobs T. (2021). CRISPR screens in plants: Approaches, guidelines, and future prospects. Plant Cell 33 (4), 794–813. doi: 10.1093/plcell/koab099 33823021 PMC8226290

[B6] Gao C. (2021). Genome engineering for crop improvement and future agriculture. Cell 184 (6), 1621–1635. doi: 10.1016/j.cell.2021.01.005 33581057

[B7] Gao X. Chen J. Dai X. Zhang D. Zhao Y. (2016). An effective strategy for reliably isolating heritable and Cas9-free arabidopsis mutants generated by CRISPR/Cas9-mediated genome editing. Plant Physiol. 171 (3), 1794–1800. doi: 10.1104/pp.16.00663 27208253 PMC4936589

[B8] Gu X. Liu L. Zhang H. (2021). Transgene-free genome editing in plants. Front. Genome Ed. 3. doi: 10.3389/fgeed.2021.805317 PMC8678605 34927134

[B9] He Y. Zhu M. Wang L. Wu J. Wang Q. Wang R.. (2018). Programmed self-elimination of the CRISPR/Cas9 construct greatly accelerates the isolation of edited and transgene-free rice plants. Mol. Plant 11 (9), 1210–1213. doi: 10.1016/j.molp.2018.05.005 29857174

[B10] He Y. Zhu M. Wang L. Wu J. Wang Q. Wang R.. (2019). Improvements of TKC technology accelerate isolation of transgene-free CRISPR/Cas9-edited rice plants. Rice Sci. 26 (2), 109–117. doi: 10.1016/j.rsci.2018.11.001

[B11] Honda C. Ohkawa K. Kusano H. Teramura H. Shimada H. (2021). A simple method for in planta tomato transformation by inoculating floral buds with a sticky *Agrobacterium tumefaciens* suspension. Plant Biotechnol. (Tokyo) 38 (1), 153–156. doi: 10.5511/plantbiotechnology.20.0707a 34177335 PMC8215470

[B12] Hwang H. Yu M. Lai E. (2017). Agrobacterium-mediated plant transformation: Biology and applications. Arabidopsis Book 15, e0186. doi: 10.1199/tab.0186 31068763 PMC6501860

[B13] Jones H. (2015). Regulatory uncertainty over genome editing. Nat. Plants 1, 14011. doi: 10.1038/nplants.2014.11 27246057

[B14] Jo J. Won S. Son D. Lee B. (2004). Paraquat resistance of transgenic tobacco plants over-expressing the ochrobactrum anthropi pqrA gene. Biotechnol. Lett. 26 (18), 1391–1396. doi: 10.1023/B:BILE.0000045638.82348.7a 15604769

[B15] Kim J. Kim J. S. (2016). Bypassing GMO regulations with CRISPR gene editing. Nat. Biotechnol. 34 (10), 1014–1015. doi: 10.1038/nbt.3680 27727209

[B16] Kong X. Pan W. Sun N. Zhang T. Liu L. Zhang H. (2021). GLABRA2-based selection efficiently enriches Cas9-generated nonchimeric mutants in the T1 generation. Plant Physiol. 187 (2), 758–768. doi: 10.1093/plphys/kiab356 34608972 PMC8491020

[B17] Kuscu C. Parlak M. Tufan T. Yang J. Szlachta K. Wei X.. (2017). CRISPR-STOP: gene silencing through base-editing-induced nonsense mutations. Nat. Methods 14, 710–712. doi: 10.1038/nmeth.4327 28581493

[B18] Liang Z. Chen K. Li T. Zhang Y. Wang Y. Zhao Q.. (2017). Efficient DNA-free genome editing of bread wheat using CRISPR/Cas9 ribonucleoprotein complexes. Nat. Commun. 8, 14261. doi: 10.1038/ncomms14261 28098143 PMC5253684

[B19] Li J. Mu J. Bai J. Fu F. Zou T. An F.. (2013). Paraquat Resistant1, a golgi-localized putative transporter protein, is involved in intracellular transport of paraquat. Plant Physiol. 162 (1), 470–483. doi: 10.1104/pp.113.213892 23471133 PMC3641224

[B20] Lu H. Liu S. Xu S. Chen W. Zhou X. Tan Y.. (2017). CRISPR-s: An active interference element for a rapid and inexpensive selection of genome-edited, transgene-free rice plants. Plant Biotechnol. J. 15 (11), 1371–1373. doi: 10.1111/pbi.12788 28688132 PMC5633759

[B21] Luo Q. Chen S. Zhu J. Ye L. Hall N. Basak S.. (2022). Overexpression of EiKCS confers paraquat-resistance in rice (*Oryza sativa* l.) by promoting the polyamine pathway. Pest Manage. Sci. 78 (1), 246–262. doi: 10.1002/ps.6628 PMC9292836 34476895

[B22] Nazish T. Huang Y. Zhang J. Xia J. Alfatih A. Luo C.. (2022). Understanding paraquat resistance mechanisms in *Arabidopsis thaliana* to facilitate the development of paraquat-resistant crops. Plant Commun. 3 (3), 100321. doi: 10.1016/j.xplc.2022.100321 35576161 PMC9251430

[B23] Park J. Choe S. (2019). DNA-free genome editing with preassembled CRISPR/Cas9 ribonucleoproteins in plants. Transgenic Res. 28 (Suppl 2), 61–64. doi: 10.1007/s11248-019-00136-3 31321685

[B24] Rinne J. Witte C. Herde M. (2021). Loss of *MAR1* function is a marker for co-selection of CRISPR-induced mutations in plants. Front. Genome Ed. 3. doi: 10.3389/fgeed.2021.723384 PMC8525433 34713265

[B25] Shamshirgaran Y. Liu J. Sumer H. Verma P. Taheri-Ghahfarokhi A. (2022). Tools for efficient genome editing; ZFN, TALEN, and CRISPR. Methods Mol. Biol. 2495, 29–46. doi: 10.1007/978-1-0716-2301-5_2 35696026

[B26] Stuttmann J. Barthel K. Martin P. Ordon J. Erickson J. Herr R.. (2021). Highly efficient multiplex editing: One-shot generation of 8x *Nicotiana benthamiana* and 12x arabidopsis mutants. Plant J. 106 (1), 8–22. doi: 10.1111/tpj.15197 33577114

[B27] Svitashev S. Schwartz C. Lenderts B. Young J. Mark Cigan A. (2016). Genome editing in maize directed by CRISPR-Cas9 ribonucleoprotein complexes. Nat. Commun. 7, 13274. doi: 10.1038/ncomms13274 27848933 PMC5116081

[B28] Thines B. Katsir L. Melotto M. Niu Y. Mandaokar A. Liu G.. (2007). JAZ repressor proteins are targets of the SCF(COI1) complex during jasmonate signalling. Nature 448 (7154), 661–665. doi: 10.1038/nature05960 17637677

[B29] Turnbull C. Lillemo M. Hvoslef-Eide T. (2021). Global regulation of genetically modified crops amid the gene edited crop boom - a review. Front. Plant Sci. 12. doi: 10.3389/fpls.2021.630396 PMC7943453 33719302

[B30] Wang C. Liu Q. Shen Y. Hua Y. Wang J. Lin J.. (2019). Clonal seeds from hybrid rice by simultaneous genome engineering of meiosis and fertilization genes. Nat. Biotechnol. 37 (3), 283–286. doi: 10.1038/s41587-018-0003-0 30610223

[B31] Wang Z. Xing H. Dong L. Zhang H. Han C. Wang X.. (2015). Egg cell-specific promoter-controlled CRISPR/Cas9 efficiently generates homozygous mutants for multiple target genes in arabidopsis in a single generation. Genome Biol. 16, 144. doi: 10.1186/s13059-015-0715-0 26193878 PMC4507317

[B32] Xia J. Nazish T. Javaid A. Ali M. Liu Q. Wang L.. (2021). A gain-of-function mutation of the MATE family transporter DTX6 confers paraquat resistance in arabidopsis. Mol. Plant 14 (12), 2126–2133. doi: 10.1016/j.molp.2021.09.004 34509638

[B33] Xing H. Dong L. Wang Z. Zhang H. Han C. Liu B.. (2014). A CRISPR/Cas9 toolkit for multiplex genome editing in plants. BMC Plant Biol. 14, 327. doi: 10.1186/s12870-014-0327-y 25432517 PMC4262988

[B34] Xu W. Yang Y. Liu Y. Kang G. Wang F. Li L.. (2020). Discriminated sgRNAs-based surrogate system greatly enhances the screening efficiency of plant base-edited cells. Mol. Plant 13 (1), 169–180. doi: 10.1016/j.molp.2019.10.007 31634585

[B35] Yu Q. Powles S. (2014). Resistance to AHAS inhibitor herbicides: Current understanding. Pest Manage. Sci. 70 (9), 1340–1350. doi: 10.1002/ps.3710 24338926

[B36] Zhang Y. Liang Z. Zong Y. Wang Y. Liu J. Chen K.. (2016a). Efficient and transgene-free genome editing in wheat through transient expression of CRISPR/Cas9 DNA or RNA. Nat. Commun. 7, 12617. doi: 10.1038/ncomms12617 27558837 PMC5007326

[B37] Zhang R. Liu J. Chai Z. Chen S. Bai Y. Zong Y.. (2019). Generation of herbicide tolerance traits and a new selectable marker in wheat using base editing. Nat. Plants 5 (5), 480–485. doi: 10.1038/s41477-019-0405-0 30988404

[B38] Zhang Z. Mao Y. Ha S. Liu W. Botella J. Zhu J. (2016b). A multiplex CRISPR/Cas9 platform for fast and efficient editing of multiple genes in arabidopsis. Plant Cell Rep. 35 (7), 1519–1533. doi: 10.1007/s00299-015-1900-z 26661595 PMC5512712

[B39] Zhang Y. Ma X. Xie X. Liu Y. (2017). CRISPR/Cas9-based genome editing in plants. Prog. Mol. Biol. Transl. Sci. 149, 133–150. doi: 10.1016/bs.pmbts.2017.03.008 28712494

[B40] Zhu H. Li C. Gao C. (2020). Applications of CRISPR-cas in agriculture and plant biotechnology. Nat. Rev. Mol. Cell Biol. 21 (11), 661–677. doi: 10.1038/s41580-020-00288-9 32973356

