# Supplementary material for: A simple and efficient strategy to produce transgene-free gene edited plants in one generation using paraquat resistant 1 as a selection marker
Source: Front Plant Sci. 2023 Jan 9;13:1051991. doi: 10.3389/fpls.2022.1051991 (PMC9888365; doi:10.3389/fpls.2022.1051991)
Supplement: Supplementary file 1 [file DataSheet_1.docx]

Supplementary Material

A simple and efficient strategy to produce transgene-free gene edited plants in one generation using *paraquat resistant 1* as a selection marker

Xiangjiu Kong^1^, Wenbo Pan^2^, Tingyu Zhang^1^, Lijing Liu^1*^ and Huawei Zhang^2,3*^

^1^The Key Laboratory of Plant Development and Environmental Adaptation Biology, Ministry of Education, School of Life Sciences, Shandong University, 266237, Qingdao, China.

^2^Peking University Institute of Advanced Agricultural Sciences, 261325, Weifang, China.

^3^Shandong Laboratory of Advanced Agricultural Sciences 261325, Weifang, China.


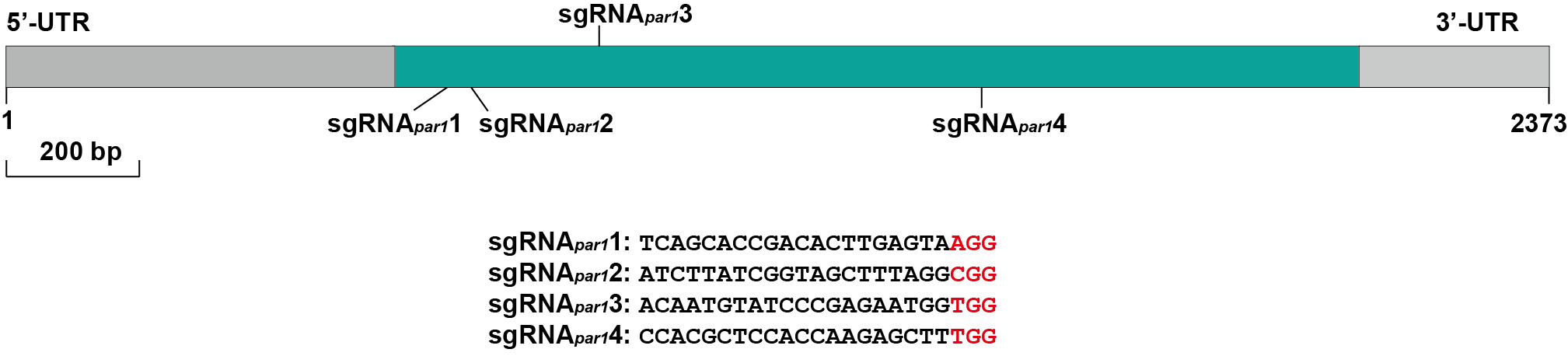


**Supplemental Figure S1.** The target sites of sgRNA*_par1_* 1-4. The structure of *PAR1* represented by exon (blue bar) and UTRs (gray bars). The sgRNA*_par1_* 1-4 target sites are indicated, and the PAM sequences are highlighted in red. UTR: untranslated region.


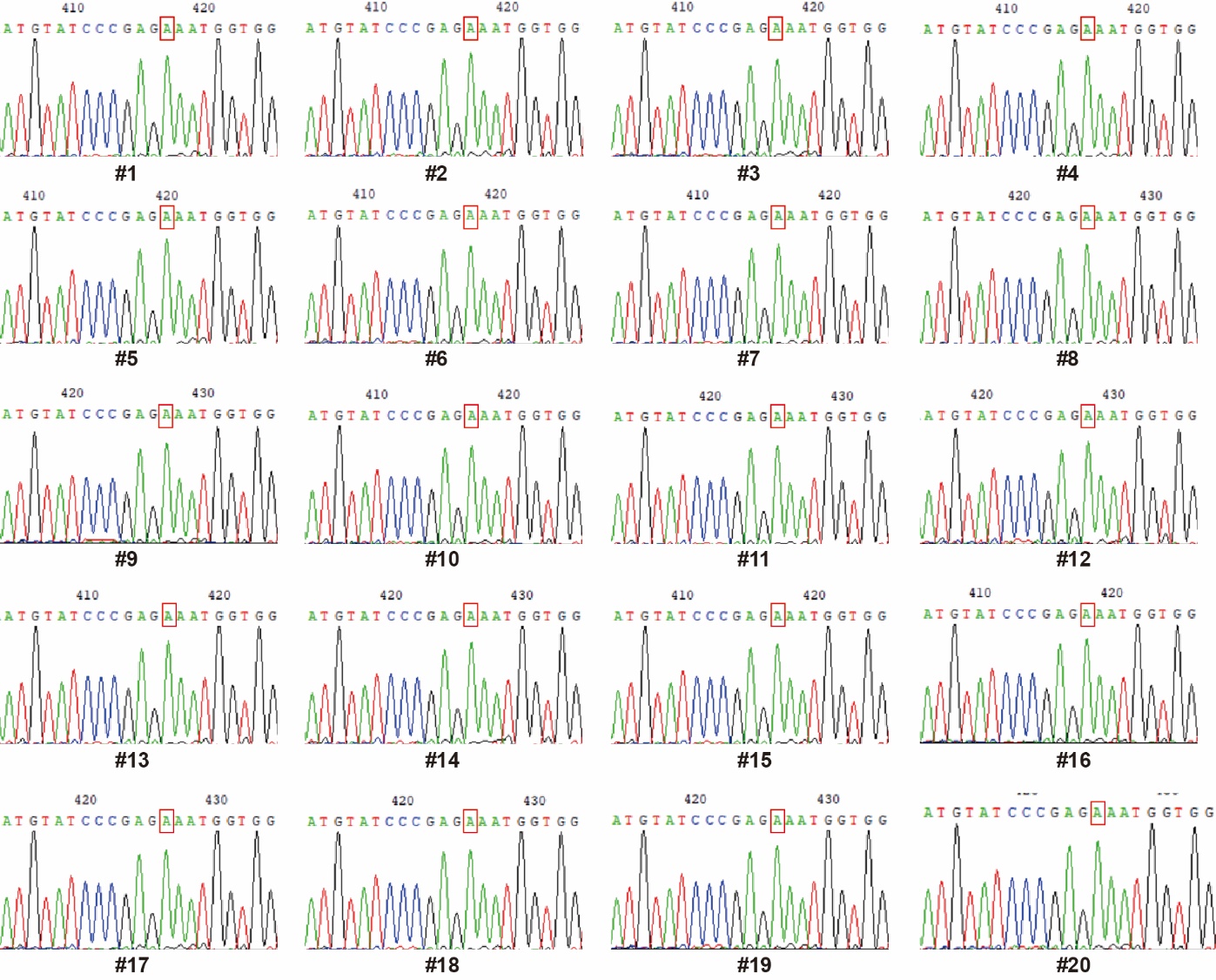


**Supplemental Figure S2.** The Sanger sequencing chromatograms for the *par1* mutants selected by paraquat. The inserted base is marked with a red box


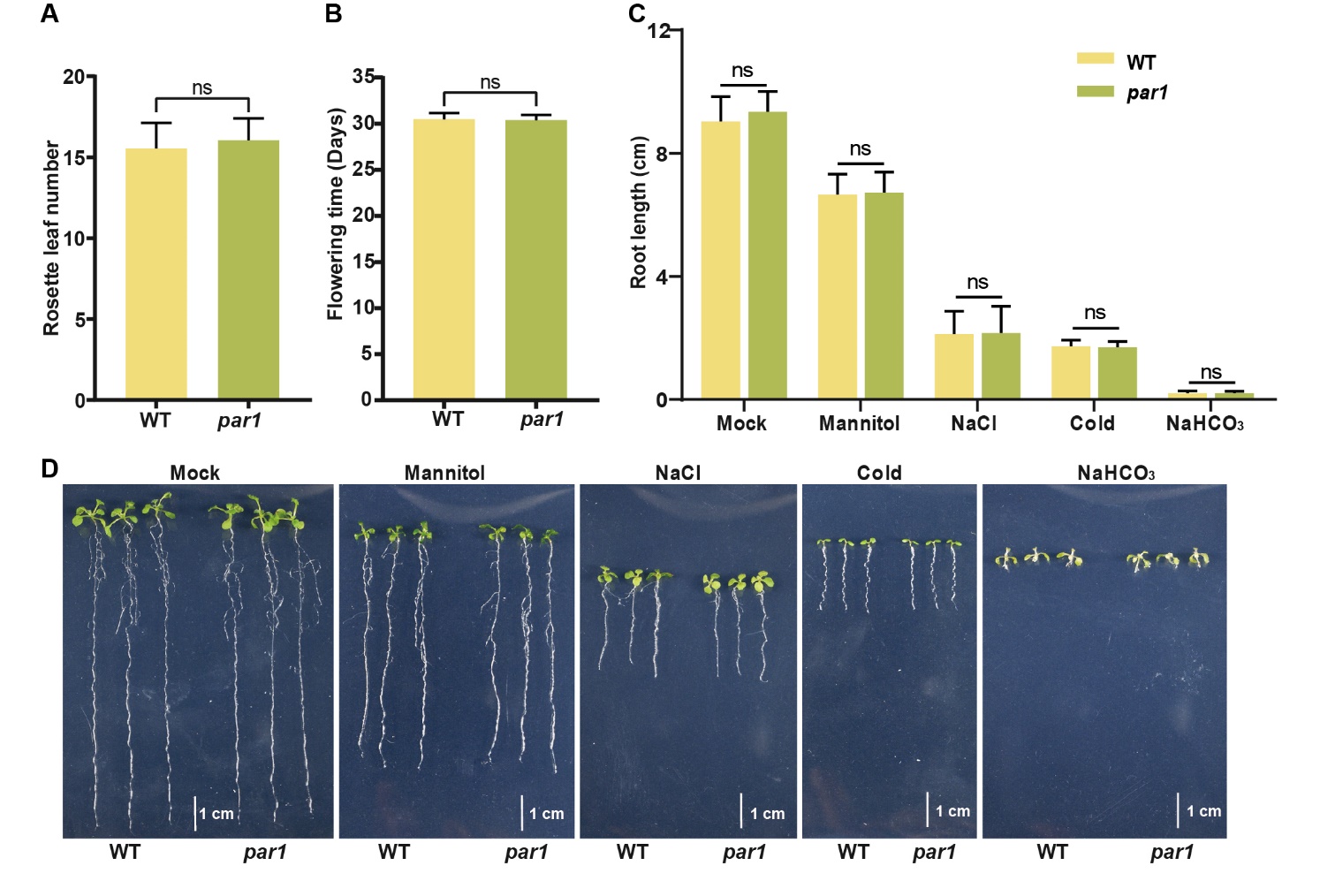


**Supplemental Figure S3. Influence of *PAR1* mutation on flowering time and stress responses in Arabidopsis.**

**(A)** Number of rosette leaves at bolting for WT and *par1* mutant (*n* = 20) under 16 h light/8 h dark photoperiod. **(B)** Flowering time for WT and *par1*mutant under 16 h light/8 h dark photoperiod (*n* = 20). **(C)** The analysis of primary root length of WT and *par1* mutant grown on 1/2 MS plates with or without 200 mM Mannitol, 150 mM NaCl or 8 mM NaHCO_3_ at 22℃ or grown on 1/2 MS plates at 12℃ (Cold) for 12 d. **(D)** Photograph of representative plants at 12 days growing under various conditions. Data are shown as mean ± SD. ns: no significant difference.


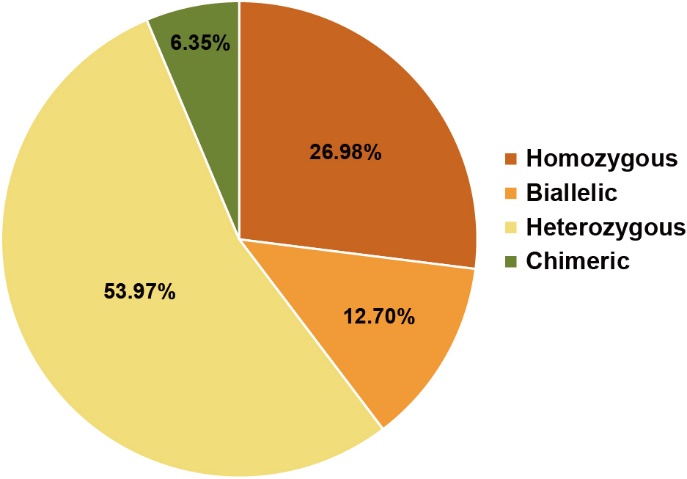


**Supplemental Figure S4.** Percentages of different types of mutations at the *PAR1* locus among all T1 plants selected on hygromycin plates.


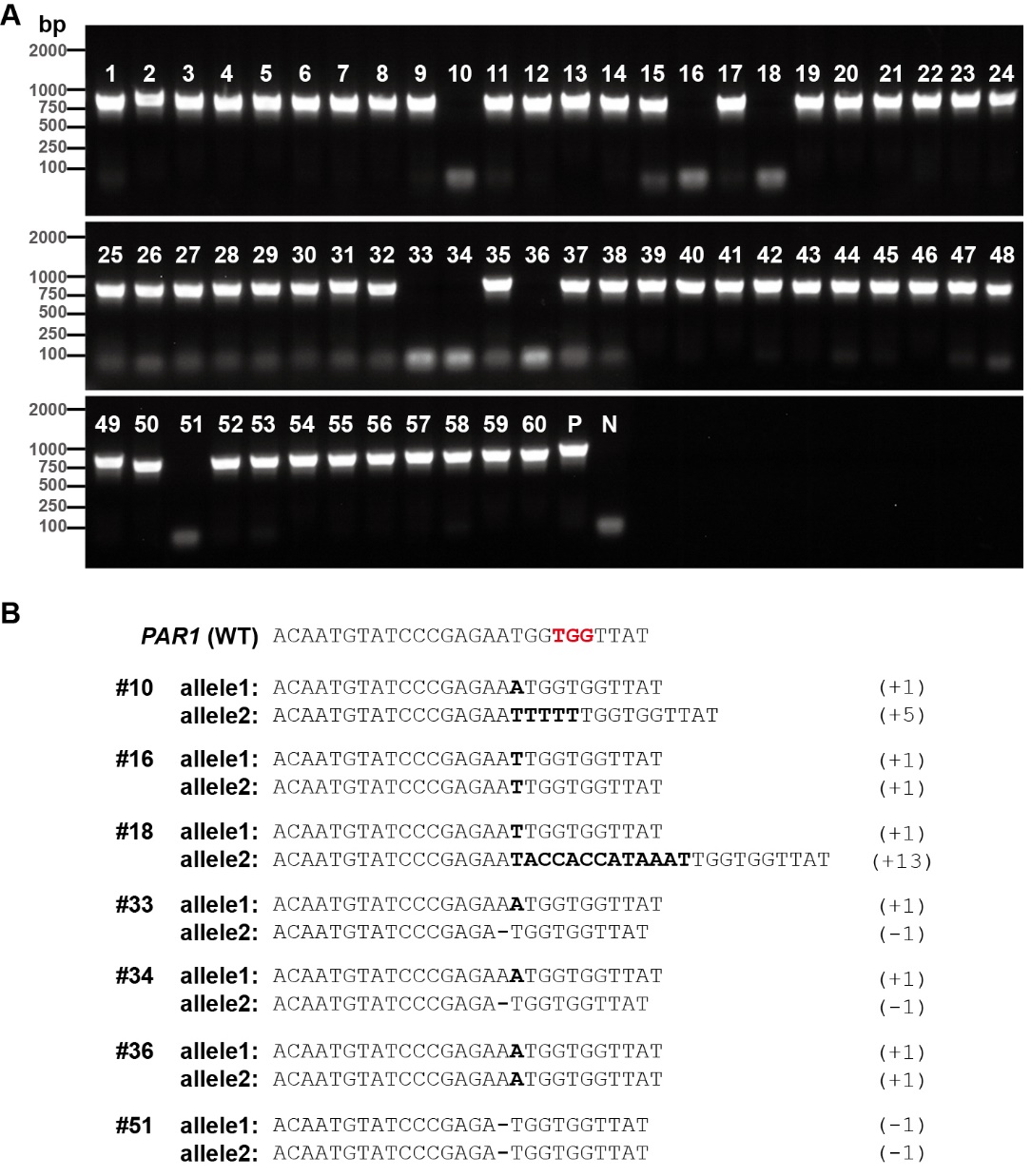


**Supplemental Figure S5.** The identification of transgene-free sgRNA*_par1_* 3 seedlings. **(A)** The PCR results of all sgRNA*_par1_* 3 T1 plants amplified with *Cas*9-specific primers. P: positive control (PCR with DNA extracted from a transgenic plant containing Cas9). N: no sample control (PCR with no DNA sample). **(B)** The DNA sequences of the transgene-free *par1* mutants. The reference sequence of WT is shown on the top. The PAM sequence is shown in red. These indels in mutant alleles are depicted by a dash (deletion) or bold letter (insertion).


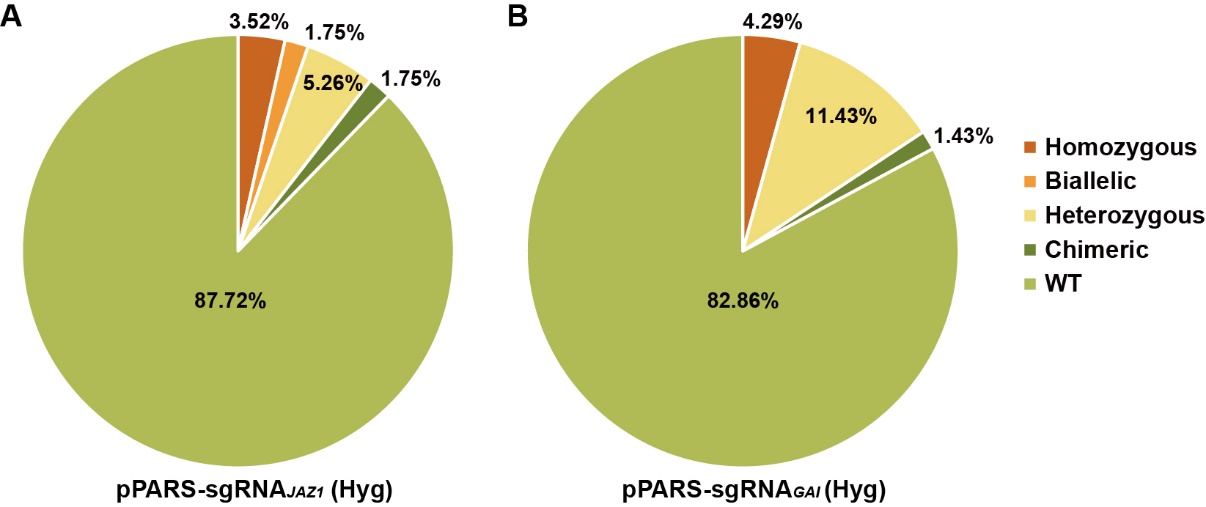


**Supplemental Figure S6.** Percentages of different types of mutations at target loci among all T1 plants selected on hygromycin (Hyg) plates. **(A)** Percentages of different types of mutations at *JAZ1* loci among all T1 plants selected on Hyg plates. **(B)** Percentages of different types of mutations at *GAI* loci among all T1 plants selected on Hyg plates.


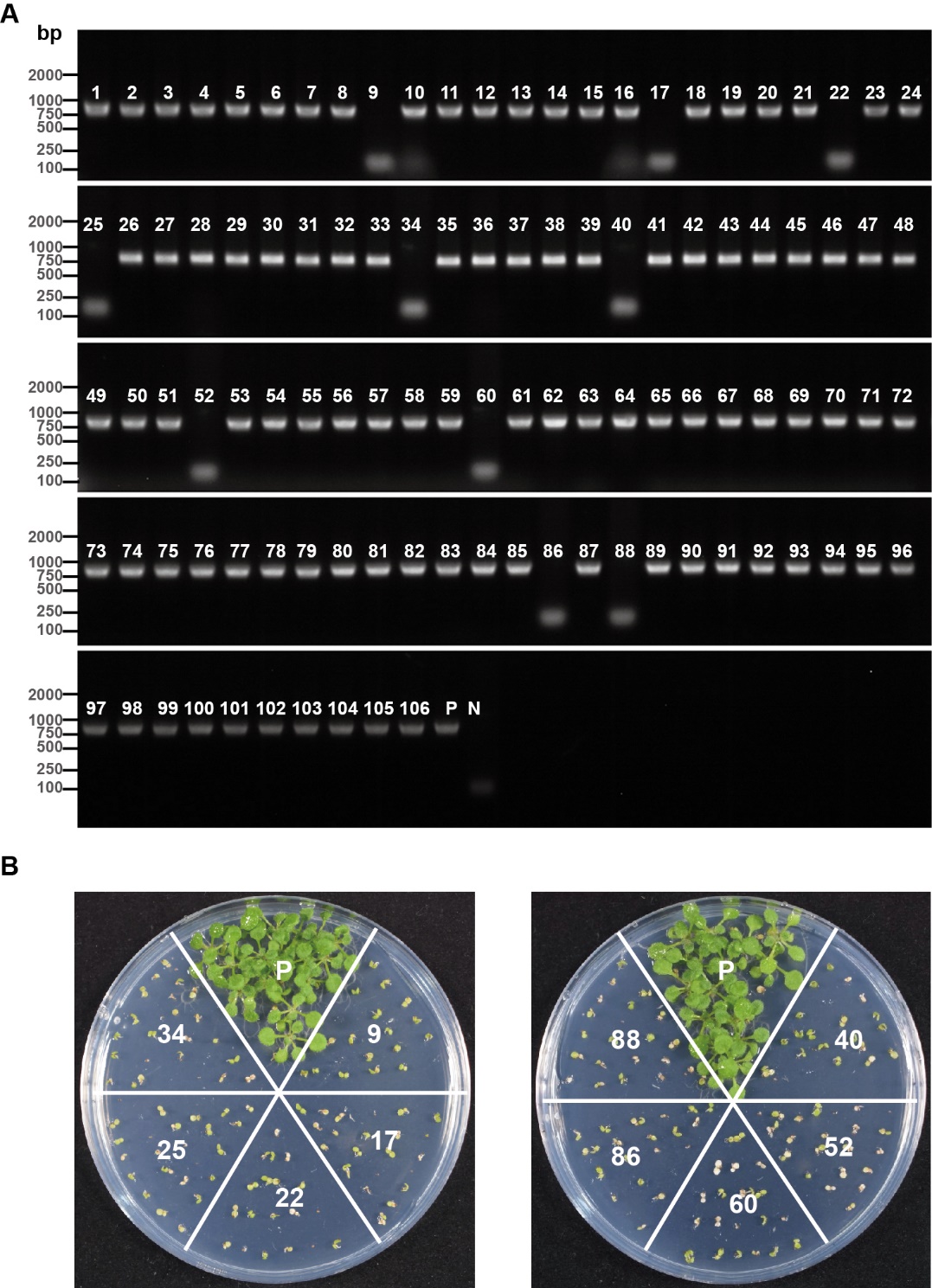


**Supplemental Figure S7.** T-DNA integration of pPARS-sgRNA*_JAZ1_* T1 plants. **(A)** PCR results of all pPARS-sgRNA*_JAZ1_* T1 plants amplified with *Cas*9-specific primers. P: positive control (PCR with DNA extracted from a transgenic plant containing Cas9). N: no sample control (PCR with no DNA sample). **(B)** The progenies of transgene-free *jaz1* mutants are sensitive to hygromycin. Seeds of these transgene-free T1 plants were sown on 1/2 MS plates supplemented with 25 mg/L hygromycin for two weeks.


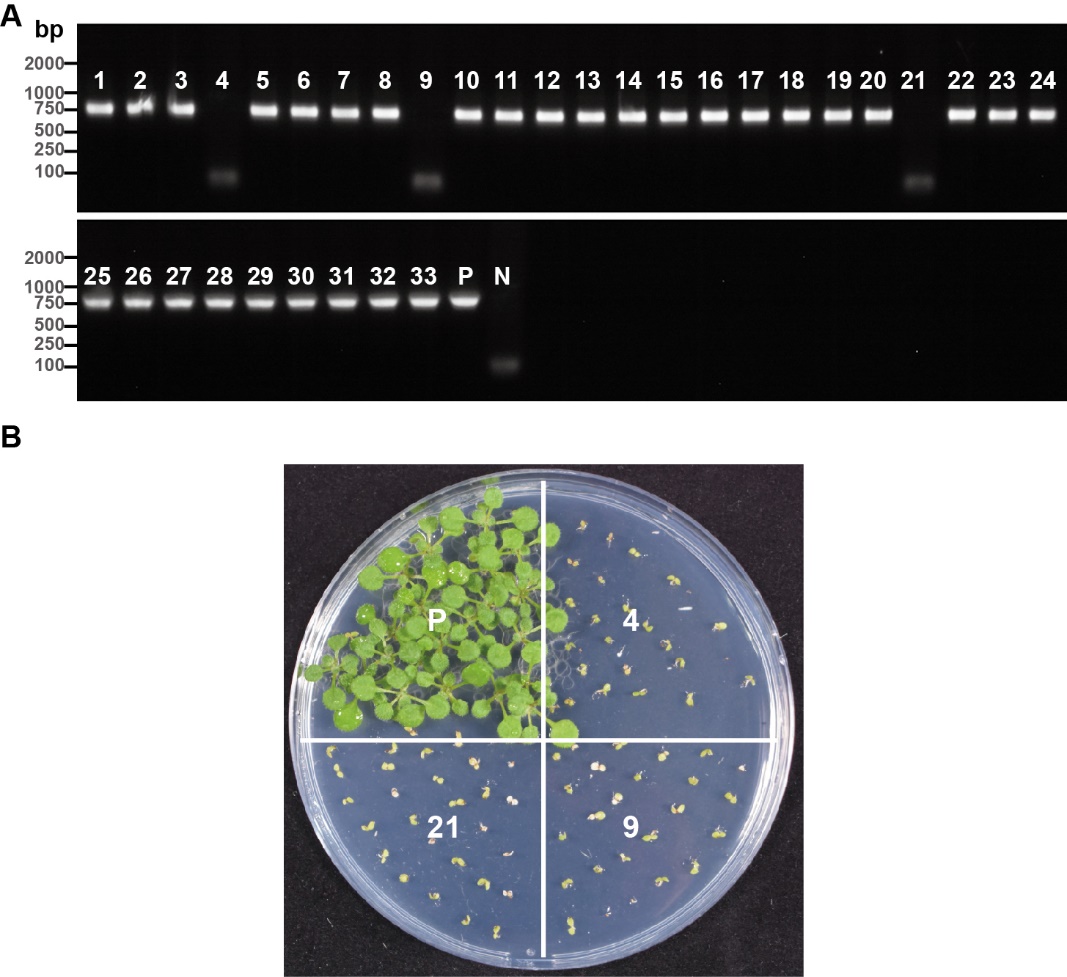


**Supplemental Figure S8.** T-DNA integration of pPARS-sgRNA*_GAI_* T1 plants. **(A)** PCR results of all pPARS-sgRNA*_GAI_* T1 plants amplified with *Cas*9-specific primers. P: positive control (PCR with DNA extracted from a transgenic plant containing Cas9). N: no sample control (PCR with no DNA sample). **(B)** The progenies of transgene-free *gai* mutants are sensitive to hygromycin. Seeds of these transgene-free T1 plants were sown on 1/2 MS plates supplemented with 25 mg/L hygromycin for two weeks.

**
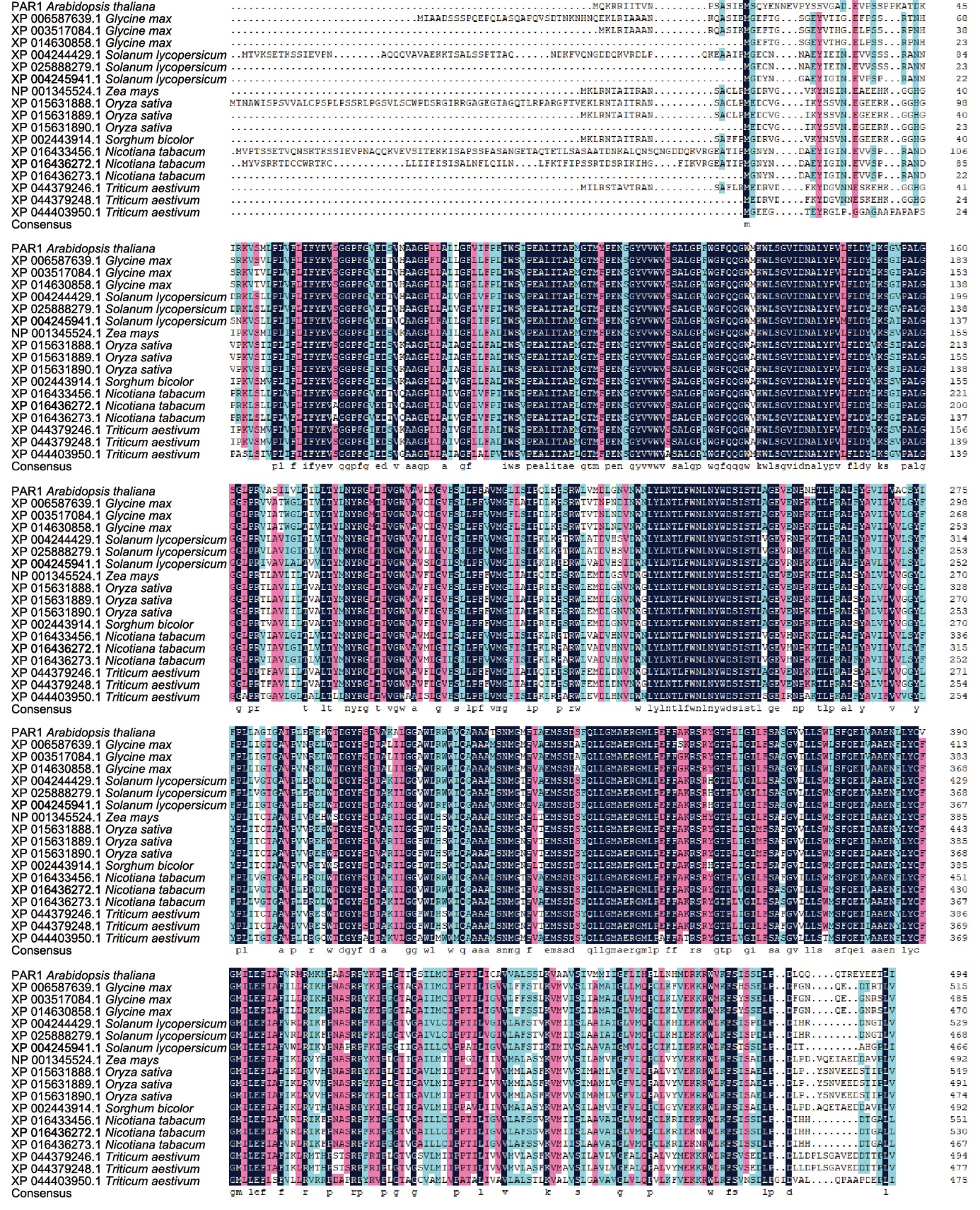
**

**Supplemental Figure S9.** Alignment of the protein sequences of the PAR1 and its homologs in different species. Amino acids identical in all sequences are highlighted with black shading. Amino acids with > 75% homology are highlighted by pink shading. Amino acids with > 50% homology are marked with light blue shading. The numbers at the right end of the alignment are the positions of corresponding protein. The letters at the bottom of the alignment are the consensus amino acid residues.

**Supplemental Table S1.** The primers used in this study.

| **Primer name** | **Primer sequence** |  |
| --- | --- | --- |
| PAR1-1-sgRNA-F | ATTGTCAGCACCGACACTTGAGTA | dsDNA for the first target of *PAR1* |
| PAR1-1-sgRNA-R | AAACTACTCAAGTGTCGGTGCTGA |  |
| PAR1-2-sgRNA-F | ATTGATCTTATCGGTAGCTTTAGG | dsDNA for the second target of *PAR1* |
| PAR1-2-sgRNA-R | AAACCCTAAAGCTACCGATAAGAT |  |
| PAR1-3-sgRNA-F | ATTGACAATGTATCCCGAGAATGG | dsDNA for the third target of *PAR1* |
| PAR1-3-sgRNA-R | AAACCCATTCTCGGGATACATTGT |  |
| PAR1-4-sgRNA-F | ATTGCCACGCTCCACCAAGAGCTT | dsDNA for the fourth target of *PAR1* |
| PAR1-4-sgRNA-R | AAACAAGCTCTTGGTGGAGCGTGG |  |
| JAZ1-sgRNA1-F | ATTGCAATAGGAAGTTCTGTCAA | dsDNA for the target of *JAZ1* |
| JAZ1-sgRNA1-R | AAACTTGACAGAACTTCCTATTG |  |
| GAI-sgRNA1-F | ATTGATGAGCTTCTAGCTGTTCT | dsDNA for the fourth target of *GAI* |
| GAI-sgRNA1-R | AAACAGAACAGCTAGAAGCTCAT |  |
| PAR1-2Tar-F | TAGAGTCGAAGTAGTGATTGTGAGACCTGAATTCAGGTCTCAGTTTTAGAGCTAGAAATAGC | construction of pPARs vector |
| PAR1-2Tar-R | GCTATTTCTAGCTCTAAAACCCATTCTCGGGATACATTGTCAATCTCTTAGTCGACTCTA |  |
| PAR1-123IF | ACAACGAACCCTAAATTCAAAAT | genotyping identification of the first three target of *PAR1* |
| PAR1-123IR | AGAAAACTCCCATAAGCACAGC |  |
| PAR1-4IF | TGACGTATCTGAACTACAGGGGA | genotyping identification of the fourth target of *PAR1* |
| PAR1-4IR | CATTCTCATTCTAACAAACGCTA |  |
| JAZ1-IF | TTGACTATATTCTACGCCGGGC | genotyping identification of the target of *JAZ1* |
| JAZ1-IR | GTAAACAAATGAAAACAAAACACACCT |  |
| GAI-IF | CCCCATCACGAGCCTTTTTAT | genotyping identification of the target of *GAI* |
| GAI-IR | CGTCGCTGTAGTGGTTTCCAC |  |
| Cas9-IF | CTCCCTAAGCACTCGCTCCTG | check the integration of Cas9 |
| Cas9-IR | TCTGCGTGGTCTGATTCTCCC |  |

**Supplemental Table S2.** The number of mutants and mutation frequencies of target genes obtained in this study.

|  | **Sample**  (number) | **Homozygous**  (number/ratio) | **Biallelic**  (number/ratio) | **Heterozygous**  (number/ratio) | **Chimeric**  (number/ratio) | **WT**  (number/ratio) |
| --- | --- | --- | --- | --- | --- | --- |
| **pHEE401E-sgRNA*_par1_*1** | 45 | 0/0 | 0/0 | 21/46.67% | 0/0 | 24/53.33% |
| **pHEE401E-sgRNA*_par1_*2** | 44 | 1/2.27% | 0/0 | 5/11.37% | 0/0 | 38/86.36% |
| **pHEE401E-sgRNA*_par1_*3** | 30 | 6/20.00% | 3/10.00% | 4/13.33% | 0/0 | 17/56.67% |
| **pHEE401E-sgRNA*_par1_*4** | 44 | 0/0 | 5/11.36% | 2/4.55% | 0/0 | 37/84.09% |
| **pHEE401E-sgRNA*_par1_*3 (PQ)** | 60 | 26/43.33% | 19/31.67% | 12/20.00% | 3/5.00% | 0/0 |
| **pHEE401E-sgRNA*_par1_*3 (Hyg)** | 63 (mutants) | 17/26.98% | 8/12.70% | 34/53.97% | 4/6.35% | 0/0 |
| **pPARS-sgRNA*_JAZ1_* (PQ)** | 25 | 6/24.00% | 8/32.00% | 3/12.00% | 1/4.00% | 7/28.00% |
| **pPARS-sgRNA*_JAZ1_* (Hyg)** | 57 | 2/3.52% | 1/1.75% | 3/5.26% | 1/1.75% | 50/87.72% |
| **pPARS-sgRNA*_GAI_* (PQ)** | 25 | 2/8.00% | 5/20.00% | 1/4.00% | 0/0 | 17/68.00% |
| **pPARS-sgRNA*_GAI_* (Hyg)** | 70 | 3/4.29% | 0/0 | 8/11.43% | 1/1.43% | 58/82.86% |
| **pHEE401E-sgRNA*_JAZ1_*** | 136 | 17/12.50% | 12/8.82% | 12/8.82% | 0/0 | 95/69.86% |
| **pHEE401E-sgRNA*_GAI_*** | 94 | 1/1.06% | 1/1.06% | 3/3.19% | 5/5.33% | 84/89.36% |

**Supplementary Data Set S1.** DNA Sequence of pPARS plasmid.

GTTTACCCGCCAATATATCCTGTCAAACACTGATAGTTTAAACTGAAGGCGGGAAACGACAATCTGATCCAAGCTCAAGCTGCTCTAGCATTCGCCATTCAGGCTGCGCAACTGTTGGGAAGGGCGATCGGTGCGGGCCTCTTCGCTATTACGCCAGCTGGCGAAAGGGGGATGTGCTGCAAGGCGATTAAGTTGGGTAACGCCAGGGTTTTCCCAGTCACGACGTTGTAAAACGACGGCCAGTGCCAAGCTTCGACTTGCCTTCCGCACAATACATCATTTCTTCTTAGCTTTTTTTCTTCTTCTTCGTTCATACAGTTTTTTTTTGTTTATCAGCTTACATTTTCTTGAACCGTAGCTTTCGTTTTCTTCTTTTTAACTTTCCATTCGGAGTTTTTGTATCTTGTTTCATAGTTTGTCCCAGGATTAGAATGATTAGGCATCGAACCTTCAAGAATTTGATTGAATAAAACATCTTCATTCTTAAGATATGAAGATAATCTTCAAAAGGCCCCTGGGAATCTGAAAGAAGAGAAGCAGGCCCATTTATATGGGAAAGAACAATAGTATTTCTTATATAGGCCCATTTAAGTTGAAAACAATCTTCAAAAGTCCCACATCGCTTAGATAAGAAAACGAAGCTGAGTTTATATACAGCTAGAGTCGAAGTAGTGATTGTGAGACCTGAATTCAGGTCTCAGTTTTAGAGCTAGAAATAGCAAGTTAAAATAAGGCTAGTCCGTTATCAACTTGAAAAAGTGGCACCGAGTCGGTGCTTTTTTTTGCAAAATTTTCCAGATCGATTTCTTCTTCCTCTGTTCTTCGGCGTTCAATTTCTGGGGTTTTCTCTTCGTTTTCTGTAACTGAAACCTAAAATTTGACCTAAAAAAAATCTCAAATAATATGATTCAGTGGTTTTGTACTTTTCAGTTAGTTGAGTTTTGCAGTTCCGATGAGATAAACCAATATTAATCCAAACTACTGCAGCCTGACAGACAAATGAGGATGCAAACAATTTTAAAGTTTATCTAACGCTAGCTGTTTTGTTTCTTCTCTCTGGTGCACCAACGACGGCGTTTTCTCAATCATAAAGAGGCTTGTTTTACTTAAGGCCAATAATGTTGATGGATCGAAAGAAGAGGGCTTTTAATAAACGAGCCCGTTTAAGCTGTAAACGATGTCAAAAACATCCCACATCGTTCAGTTGAAAATAGAAGCTCTGTTTATATATTGGTAGAGTCGACTAAGAGATTGACAATGTATCCCGAGAATGGGTTTTAGAGCTAGAAATAGCAAGTTAAAATAAGGCTAGTCCGTTATCAACTTGAAAAAGTGGCACCGAGTCGGTGCTTTTTTTTGCAAAATTTTCCAGATCGATTTCTTCTTCCTCTGTTCTTCGGCGTTCAATTTCTGGGGTTTTCTCTTCGTTTTCTGTAACTGAAACCTAAAATTTGACCTAAAAAAAATCTCAAATAATATGATTCAGTGGTTTTGTACTTTTCAGTTAGTTGAGTTTTGCAGTTCCGATGAGATAAACCAATACCATGGTTATACTAGTGAATAAAAGCATTTGCGTTTGGTTTATCATTGCGTTTATACAAGGACAGAGATCCACTGAGCTGGAATAGCTTAAAACCATTATCAGAACAAAATAAACCATTTTTTGTTAAGAATCAGAGCATAGTAAACAACAGAAACAACCTAAGAGAGGTAACTTGTCCAAGAAGATAGCTAATTATATCTATTTTATAAAAGTTATCATAGTTTGTAAGTCACAAAAGATGCAAATAACAGAGAAACTAGGAGACTTGAGAATATACATTCTTGTATATTTGTATTCGAGATTGTGAAAATTTGACCATAAGTTTAAATTCTTAAAAAGATATATCTGATCTAGGTGATGGTTATAGACTGTAATTTTACCACATGTTTAATGATGGATAGTGACACACATGACACATCGACAACACTATAGCATCTTATTTAGATTACAACATGAAATTTTTCTGTAATACATGTCTTTGTACATAATTTAAAAGTAATTCCTAAGAAATATATTTATACAAGGAGTTTAAAGAAAACATAGCATAAAGTTCAATGAGTAGTAAAAACCATATACAGTATATAGCATAAAGTTCAATGAGTTTATTACAAAAGCATTGGTTCACTTTCTGTAACACGACGTTAAACCTTCGTCTCCAATAGGAGCGCTACTGATTCAACATGCCAATATATACTAAATACGTTTCTACAGTCAAATGCTTTAACGTTTCATGATTAAGTGACTATTTACCGTCAATCCTTTCCCATTCCTCCCACTAATCCAACTTTTTAATTACTCTTAAATCACCACTAAGCTAGTAACGCCTATCATGAATTAGCTCTACTAAATCTAGCAACCTTTCAAATTTGCAGTATTGCAGGTGTCTCTGTGTCTTTAAAATAGTTGCCTTATGATTTCTTCGGTTTCAAGATGATCAAATAGTTATAGATTTCATGCTCACACATGCTCATTAGATGTGTACATACTTTACTTACCCAAATCTATTTTCTCGCAAAGATTTTGATGGTAAAGCTGATTTGGTTCTATTGAACTAAATCAAACGAGTTTCAGACTGAGTGATTCTAATCCGGCCCATTAGCCCCTAAACAGACCCACTAATTACGCAGCTTTTAATAGAGTAATTACACCTAGTTTACCCACTAAACCACTAAGCACTAATTATCTCACAATCTAATGAGCTTCCCTCGTAATTACTTGGGCTTTCACTCTACCATTTATTTGTAACAGTCAAGTCTCTACTGTCTCTATATAAACTCTCTAAAGTTAACACACAATTCTCATCACAAACAAATCAACCAAAGCAACTTCTACTCTTTCTTCTTTCGACCTTATCAATCTGTTGAGAAATCTAGATGGATTACAAGGACCACGACGGGGATTACAAGGACCACGACATTGATTACAAGGATGATGATGACAAGATGGCTCCGAAGAAGAAGAGGAAGGTTGGCATCCACGGGGTGCCAGCTGCTGACAAGAAGTACTCGATCGGCCTCGATATTGGGACTAACTCTGTTGGCTGGGCCGTGATCACCGACGAGTACAAGGTGCCCTCAAAGAAGTTCAAGGTCCTGGGCAACACCGATCGGCATTCCATCAAGAAGAATCTCATTGGCGCTCTCCTGTTCGACAGCGGCGAGACGGCTGAGGCTACGCGGCTCAAGCGCACCGCCCGCAGGCGGTACACGCGCAGGAAGAATCGCATCTGCTACCTGCAGGAGATTTTCTCCAACGAGATGGCGAAGGTTGACGATTCTTTCTTCCACAGGCTGGAGGAGTCATTCCTCGTGGAGGAGGATAAGAAGCACGAGCGGCATCCAATCTTCGGCAACATTGTCGACGAGGTTGCCTACCACGAGAAGTACCCTACGATCTACCATCTGCGGAAGAAGCTCGTGGACTCCACAGATAAGGCGGACCTCCGCCTGATCTACCTCGCTCTGGCCCACATGATTAAGTTCAGGGGCCATTTCCTGATCGAGGGGGATCTCAACCCGGACAATAGCGATGTTGACAAGCTGTTCATCCAGCTCGTGCAGACGTACAACCAGCTCTTCGAGGAGAACCCCATTAATGCGTCAGGCGTCGACGCGAAGGCTATCCTGTCCGCTAGGCTCTCGAAGTCTCGGCGCCTCGAGAACCTGATCGCCCAGCTGCCGGGCGAGAAGAAGAACGGCCTGTTCGGGAATCTCATTGCGCTCAGCCTGGGGCTCACGCCCAACTTCAAGTCGAATTTCGATCTCGCTGAGGACGCCAAGCTGCAGCTCTCCAAGGACACATACGACGATGACCTGGATAACCTCCTGGCCCAGATCGGCGATCAGTACGCGGACCTGTTCCTCGCTGCCAAGAATCTGTCGGACGCCATCCTCCTGTCTGATATTCTCAGGGTGAACACCGAGATTACGAAGGCTCCGCTCTCAGCCTCCATGATCAAGCGCTACGACGAGCACCATCAGGATCTGACCCTCCTGAAGGCGCTGGTCAGGCAGCAGCTCCCCGAGAAGTACAAGGAGATCTTCTTCGATCAGTCGAAGAACGGCTACGCTGGGTACATTGACGGCGGGGCCTCTCAGGAGGAGTTCTACAAGTTCATCAAGCCGATTCTGGAGAAGATGGACGGCACGGAGGAGCTGCTGGTGAAGCTCAATCGCGAGGACCTCCTGAGGAAGCAGCGGACATTCGATAACGGCAGCATCCCACACCAGATTCATCTCGGGGAGCTGCACGCTATCCTGAGGAGGCAGGAGGACTTCTACCCTTTCCTCAAGGATAACCGCGAGAAGATCGAGAAGATTCTGACTTTCAGGATCCCGTACTACGTCGGCCCACTCGCTAGGGGCAACTCCCGCTTCGCTTGGATGACCCGCAAGTCAGAGGAGACGATCACGCCGTGGAACTTCGAGGAGGTGGTCGACAAGGGCGCTAGCGCTCAGTCGTTCATCGAGAGGATGACGAATTTCGACAAGAACCTGCCAAATGAGAAGGTGCTCCCTAAGCACTCGCTCCTGTACGAGTACTTCACAGTCTACAACGAGCTGACTAAGGTGAAGTATGTGACCGAGGGCATGAGGAAGCCGGCTTTCCTGTCTGGGGAGCAGAAGAAGGCCATCGTGGACCTCCTGTTCAAGACCAACCGGAAGGTCACGGTTAAGCAGCTCAAGGAGGACTACTTCAAGAAGATTGAGTGCTTCGATTCGGTCGAGATCTCTGGCGTTGAGGACCGCTTCAACGCCTCCCTGGGGACCTACCACGATCTCCTGAAGATCATTAAGGATAAGGACTTCCTGGACAACGAGGAGAATGAGGATATCCTCGAGGACATTGTGCTGACACTCACTCTGTTCGAGGACCGGGAGATGATCGAGGAGCGCCTGAAGACTTACGCCCATCTCTTCGATGACAAGGTCATGAAGCAGCTCAAGAGGAGGAGGTACACCGGCTGGGGGAGGCTGAGCAGGAAGCTCATCAACGGCATTCGGGACAAGCAGTCCGGGAAGACGATCCTCGACTTCCTGAAGAGCGATGGCTTCGCGAACCGCAATTTCATGCAGCTGATTCACGATGACAGCCTCACATTCAAGGAGGATATCCAGAAGGCTCAGGTGAGCGGCCAGGGGGACTCGCTGCACGAGCATATCGCGAACCTCGCTGGCTCGCCAGCTATCAAGAAGGGGATTCTGCAGACCGTGAAGGTTGTGGACGAGCTGGTGAAGGTCATGGGCAGGCACAAGCCTGAGAACATCGTCATTGAGATGGCCCGGGAGAATCAGACCACGCAGAAGGGCCAGAAGAACTCACGCGAGAGGATGAAGAGGATCGAGGAGGGCATTAAGGAGCTGGGGTCCCAGATCCTCAAGGAGCACCCGGTGGAGAACACGCAGCTGCAGAATGAGAAGCTCTACCTGTACTACCTCCAGAATGGCCGCGATATGTATGTGGACCAGGAGCTGGATATTAACAGGCTCAGCGATTACGACGTCGATCATATCGTTCCACAGTCATTCCTGAAGGATGACTCCATTGACAACAAGGTCCTCACCAGGTCGGACAAGAACCGGGGCAAGTCTGATAATGTTCCTTCAGAGGAGGTCGTTAAGAAGATGAAGAACTACTGGCGCCAGCTCCTGAATGCCAAGCTGATCACGCAGCGGAAGTTCGATAACCTCACAAAGGCTGAGAGGGGCGGGCTCTCTGAGCTGGACAAGGCGGGCTTCATCAAGAGGCAGCTGGTCGAGACACGGCAGATCACTAAGCACGTTGCGCAGATTCTCGACTCACGGATGAACACTAAGTACGATGAGAATGACAAGCTGATCCGCGAGGTGAAGGTCATCACCCTGAAGTCAAAGCTCGTCTCCGACTTCAGGAAGGATTTCCAGTTCTACAAGGTTCGGGAGATCAACAATTACCACCATGCCCATGACGCGTACCTGAACGCGGTGGTCGGCACAGCTCTGATCAAGAAGTACCCAAAGCTCGAGAGCGAGTTCGTGTACGGGGACTACAAGGTTTACGATGTGAGGAAGATGATCGCCAAGTCGGAGCAGGAGATTGGCAAGGCTACCGCCAAGTACTTCTTCTACTCTAACATTATGAATTTCTTCAAGACAGAGATCACTCTGGCCAATGGCGAGATCCGGAAGCGCCCCCTCATCGAGACGAACGGCGAGACGGGGGAGATCGTGTGGGACAAGGGCAGGGATTTCGCGACCGTCAGGAAGGTTCTCTCCATGCCACAAGTGAATATCGTCAAGAAGACAGAGGTCCAGACTGGCGGGTTCTCTAAGGAGTCAATTCTGCCTAAGCGGAACAGCGACAAGCTCATCGCCCGCAAGAAGGACTGGGATCCGAAGAAGTACGGCGGGTTCGACAGCCCCACTGTGGCCTACTCGGTCCTGGTTGTGGCGAAGGTTGAGAAGGGCAAGTCCAAGAAGCTCAAGAGCGTGAAGGAGCTGCTGGGGATCACGATTATGGAGCGCTCCAGCTTCGAGAAGAACCCGATCGATTTCCTGGAGGCGAAGGGCTACAAGGAGGTGAAGAAGGACCTGATCATTAAGCTCCCCAAGTACTCACTCTTCGAGCTGGAGAACGGCAGGAAGCGGATGCTGGCTTCCGCTGGCGAGCTGCAGAAGGGGAACGAGCTGGCTCTGCCGTCCAAGTATGTGAACTTCCTCTACCTGGCCTCCCACTACGAGAAGCTCAAGGGCAGCCCCGAGGACAACGAGCAGAAGCAGCTGTTCGTCGAGCAGCACAAGCATTACCTCGACGAGATCATTGAGCAGATTTCCGAGTTCTCCAAGCGCGTGATCCTGGCCGACGCGAATCTGGATAAGGTCCTCTCCGCGTACAACAAGCACCGCGACAAGCCAATCAGGGAGCAGGCTGAGAATATCATTCATCTCTTCACCCTGACGAACCTCGGCGCCCCTGCTGCTTTCAAGTACTTCGACACAACTATCGATCGCAAGAGGTACACAAGCACTAAGGAGGTCCTGGACGCGACCCTCATCCACCAGTCGATTACCGGCCTCTACGAGACGCGCATCGACCTGTCTCAGCTCGGGGGCGACAAGCGGCCAGCGGCGACGAAGAAGGCGGGGCAGGCGAAGAAGAAGAAGTGAGCTCAGAGCTTTCGTTCGTATCATCGGTTTCGACAACGTTCGTCAAGTTCAATGCATCAGTTTCATTGCGCACACACCAGAATCCTACTGAGTTTGAGTATTATGGCATTGGGAAAACTGTTTTTCTTGTACCATTTGTTGTGCTTGTAATTTACTGTGTTTTTTATTCGGTTTTCGCTATCGAACTGTGAAATGGAAATGGATGGAGAAGAGTTAATGAATGATATGGTCCTTTTGTTCATTCTCAAATTAATATTATTTGTTTTTTCTCTTATTTGTTGTGTGTTGAATTTGAAATTATAAGAGATATGCAAACATTTTGTTTTGAGTAAAAATGTGTCAAATCGTGGCCTCTAATGACCGAAGTTAATATGAGGAGTAAAACACTTGTAGTTGTACCATTATGCTTATTCACTAGGCAACAAATATATTTTCAGACCTAGAAAAGCTGCAAATGTTACTGAATACAAGTATGTCCTCTTGTGTTTTAGACATTTATGAACTTTCCTTTATGTAATTTTCCAGAATCCTTGTCAGATTCTAATCATTGCTTTATAATTATAGTTATACTCATGGATTTGTAGTTGAGTATGAAAATATTTTTTAATGCATTTTATGACTTGCCAATTGATTGACAACGAATTCGTAATCATGTCATAGCTGTTTCCTGTGTGAAATTGTTATCCGCTCACAATTCCACACAACATACGAGCCGGAAGCATAAAGTGTAAAGCCTGGGGTGCCTAATGAGTGAGCTAACTCACATTAATTGCGTTGCGCTCACTGCCCGCTTTCCAGTCGGGAAACCTGTCGTGCCAGCTGCATTAATGAATCGGCCAACGCGCGGGGAGAGGCGGTTTGCGTATTGGCTAGAGCAGCTTGCCAACATGGTGGAGCACGACACTCTCGTCTACTCCAAGAATATCAAAGATACAGTCTCAGAAGACCAAAGGGCTATTGAGACTTTTCAACAAAGGGTAATATCGGGAAACCTCCTCGGATTCCATTGCCCAGCTATCTGTCACTTCATCAAAAGGACAGTAGAAAAGGAAGGTGGCACCTACAAATGCCATCATTGCGATAAAGGAAAGGCTATCGTTCAAGATGCCTCTGCCGACAGTGGTCCCAAAGATGGACCCCCACCCACGAGGAGCATCGTGGAAAAAGAAGACGTTCCAACCACGTCTTCAAAGCAAGTGGATTGATGTGAACATGGTGGAGCACGACACTCTCGTCTACTCCAAGAATATCAAAGATACAGTCTCAGAAGACCAAAGGGCTATTGAGACTTTTCAACAAAGGGTAATATCGGGAAACCTCCTCGGATTCCATTGCCCAGCTATCTGTCACTTCATCAAAAGGACAGTAGAAAAGGAAGGTGGCACCTACAAATGCCATCATTGCGATAAAGGAAAGGCTATCGTTCAAGATGCCTCTGCCGACAGTGGTCCCAAAGATGGACCCCCACCCACGAGGAGCATCGTGGAAAAAGAAGACGTTCCAACCACGTCTTCAAAGCAAGTGGATTGATGTGATATCTCCACTGACGTAAGGGATGACGCACAATCCCACTATCCTTCGCAAGACCCTTCCTCTATATAAGGAAGTTCATTTCATTTGGAGAGGACACGCTGAAATCACCAGTCTCTCTCTACAAATCTATCTCTCTCGAGCTTTCGCAGATCCGGGGGGCAATGAGATATGAAAAAGCCTGAACTCACCGCGACGTCTGTCGAGAAGTTTCTGATCGAAAAGTTCGACAGCGTCTCCGACCTGATGCAGCTCTCGGAGGGCGAAGAATCTCGTGCTTTCAGCTTCGATGTAGGAGGGCGTGGATATGTCCTGCGGGTAAATAGCTGCGCCGATGGTTTCTACAAAGATCGTTATGTTTATCGGCACTTTGCATCGGCCGCGCTCCCGATTCCGGAAGTGCTTGACATTGGGGAGTTTAGCGAGAGCCTGACCTATTGCATCTCCCGCCGTTCACAGGGTGTCACGTTGCAAGACCTGCCTGAAACCGAACTGCCCGCTGTTCTACAACCGGTCGCGGAGGCTATGGATGCGATCGCTGCGGCCGATCTTAGCCAGACGAGCGGGTTCGGCCCATTCGGACCGCAAGGAATCGGTCAATACACTACATGGCGTGATTTCATATGCGCGATTGCTGATCCCCATGTGTATCACTGGCAAACTGTGATGGACGACACCGTCAGTGCGTCCGTCGCGCAGGCTCTCGATGAGCTGATGCTTTGGGCCGAGGACTGCCCCGAAGTCCGGCACCTCGTGCACGCGGATTTCGGCTCCAACAATGTCCTGACGGACAATGGCCGCATAACAGCGGTCATTGACTGGAGCGAGGCGATGTTCGGGGATTCCCAATACGAGGTCGCCAACATCTTCTTCTGGAGGCCGTGGTTGGCTTGTATGGAGCAGCAGACGCGCTACTTCGAGCGGAGGCATCCGGAGCTTGCAGGATCGCCACGACTCCGGGCGTATATGCTCCGCATTGGTCTTGACCAACTCTATCAGAGCTTGGTTGACGGCAATTTCGATGATGCAGCTTGGGCGCAGGGTCGATGCGACGCAATCGTCCGATCCGGAGCCGGGACTGTCGGGCGTACACAAATCGCCCGCAGAAGCGCGGCCGTCTGGACCGATGGCTGTGTAGAAGTACTCGCCGATAGTGGAAACCGACGCCCCAGCACTCGTCCGAGGGCAAAGAAATAGAGTAGATGCCGACCGGGATCTGTCGATCGACAAGCTCGAGTTTCTCCATAATAATGTGTGAGTAGTTCCCAGATAAGGGAATTAGGGTTCCTATAGGGTTTCGCTCATGTGTTGAGCATATAAGAAACCCTTAGTATGTATTTGTATTTGTAAAATACTTCTATCAATAAAATTTCTAATTCCTAAAACCAAAATCCAGTACTAAAATCCAGATCCCCCGAATTAATTCGGCGTTAATTCAGTACATTAAAAACGTCCGCAATGTGTTATTAAGTTGTCTAAGCGTCAATTTGTTTACACCACAATATATCCTGCCACCAGCCAGCCAACAGCTCCCCGACCGGCAGCTCGGCACAAAATCACCACTCGATACAGGCAGCCCATCAGTCCGGGACGGCGTCAGCGGGAGAGCCGTTGTAAGGCGGCAGACTTTGCTCATGTTACCGATGCTATTCGGAAGAACGGCAACTAAGCTGCCGGGTTTGAAACACGGATGATCTCGCGGAGGGTAGCATGTTGATTGTAACGATGACAGAGCGTTGCTGCCTGTGATCACCGCGGTTTCAAAATCGGCTCCGTCGATACTATGTTATACGCCAACTTTGAAAACAACTTTGAAAAAGCTGTTTTCTGGTATTTAAGGTTTTAGAATGCAAGGAACAGTGAATTGGAGTTCGTCTTGTTATAATTAGCTTCTTGGGGTATCTTTAAATACTGTAGAAAAGAGGAAGGAAATAATAAATGGCTAAAATGAGAATATCACCGGAATTGAAAAAACTGATCGAAAAATACCGCTGCGTAAAAGATACGGAAGGAATGTCTCCTGCTAAGGTATATAAGCTGGTGGGAGAAAATGAAAACCTATATTTAAAAATGACGGACAGCCGGTATAAAGGGACCACCTATGATGTGGAACGGGAAAAGGACATGATGCTATGGCTGGAAGGAAAGCTGCCTGTTCCAAAGGTCCTGCACTTTGAACGGCATGATGGCTGGAGCAATCTGCTCATGAGTGAGGCCGATGGCGTCCTTTGCTCGGAAGAGTATGAAGATGAACAAAGCCCTGAAAAGATTATCGAGCTGTATGCGGAGTGCATCAGGCTCTTTCACTCCATCGACATATCGGATTGTCCCTATACGAATAGCTTAGACAGCCGCTTAGCCGAATTGGATTACTTACTGAATAACGATCTGGCCGATGTGGATTGCGAAAACTGGGAAGAAGACACTCCATTTAAAGATCCGCGCGAGCTGTATGATTTTTTAAAGACGGAAAAGCCCGAAGAGGAACTTGTCTTTTCCCACGGCGACCTGGGAGACAGCAACATCTTTGTGAAAGATGGCAAAGTAAGTGGCTTTATTGATCTTGGGAGAAGCGGCAGGGCGGACAAGTGGTATGACATTGCCTTCTGCGTCCGGTCGATCAGGGAGGATATCGGGGAAGAACAGTATGTCGAGCTATTTTTTGACTTACTGGGGATCAAGCCTGATTGGGAGAAAATAAAATATTATATTTTACTGGATGAATTGTTTTAGTACCTAGAATGCATGACCAAAATCCCTTAACGTGAGTTTTCGTTCCACTGAGCGTCAGACCCCGTAGAAAAGATCAAAGGATCTTCTTGAGATCCTTTTTTTCTGCGCGTAATCTGCTGCTTGCAAACAAAAAAACCACCGCTACCAGCGGTGGTTTGTTTGCCGGATCAAGAGCTACCAACTCTTTTTCCGAAGGTAACTGGCTTCAGCAGAGCGCAGATACCAAATACTGTCCTTCTAGTGTAGCCGTAGTTAGGCCACCACTTCAAGAACTCTGTAGCACCGCCTACATACCTCGCTCTGCTAATCCTGTTACCAGTGGCTGCTGCCAGTGGCGATAAGTCGTGTCTTACCGGGTTGGACTCAAGACGATAGTTACCGGATAAGGCGCAGCGGTCGGGCTGAACGGGGGGTTCGTGCACACAGCCCAGCTTGGAGCGAACGACCTACACCGAACTGAGATACCTACAGCGTGAGCTATGAGAAAGCGCCACGCTTCCCGAAGGGAGAAAGGCGGACAGGTATCCGGTAAGCGGCAGGGTCGGAACAGGAGAGCGCACGAGGGAGCTTCCAGGGGGAAACGCCTGGTATCTTTATAGTCCTGTCGGGTTTCGCCACCTCTGACTTGAGCGTCGATTTTTGTGATGCTCGTCAGGGGGGCGGAGCCTATGGAAAAACGCCAGCAACGCGGCCTTTTTACGGTTCCTGGCCTTTTGCTGGCCTTTTGCTCACATGTTCTTTCCTGCGTTATCCCCTGATTCTGTGGATAACCGTATTACCGCCTTTGAGTGAGCTGATACCGCTCGCCGCAGCCGAACGACCGAGCGCAGCGAGTCAGTGAGCGAGGAAGCGGAAGAGCGCCTGATGCGGTATTTTCTCCTTACGCATCTGTGCGGTATTTCACACCGCATATGGTGCACTCTCAGTACAATCTGCTCTGATGCCGCATAGTTAAGCCAGTATACACTCCGCTATCGCTACGTGACTGGGTCATGGCTGCGCCCCGACACCCGCCAACACCCGCTGACGCGCCCTGACGGGCTTGTCTGCTCCCGGCATCCGCTTACAGACAAGCTGTGACCGTCTCCGGGAGCTGCATGTGTCAGAGGTTTTCACCGTCATCACCGAAACGCGCGAGGCAGGGTGCCTTGATGTGGGCGCCGGCGGTCGAGTGGCGACGGCGCGGCTTGTCCGCGCCCTGGTAGATTGCCTGGCCGTAGGCCAGCCATTTTTGAGCGGCCAGCGGCCGCGATAGGCCGACGCGAAGCGGCGGGGCGTAGGGAGCGCAGCGACCGAAGGGTAGGCGCTTTTTGCAGCTCTTCGGCTGTGCGCTGGCCAGACAGTTATGCACAGGCCAGGCGGGTTTTAAGAGTTTTAATAAGTTTTAAAGAGTTTTAGGCGGAAAAATCGCCTTTTTTCTCTTTTATATCAGTCACTTACATGTGTGACCGGTTCCCAATGTACGGCTTTGGGTTCCCAATGTACGGGTTCCGGTTCCCAATGTACGGCTTTGGGTTCCCAATGTACGTGCTATCCACAGGAAACAGACCTTTTCGACCTTTTTCCCCTGCTAGGGCAATTTGCCCTAGCATCTGCTCCGTACATTAGGAACCGGCGGATGCTTCGCCCTCGATCAGGTTGCGGTAGCGCATGACTAGGATCGGGCCAGCCTGCCCCGCCTCCTCCTTCAAATCGTACTCCGGCAGGTCATTTGACCCGATCAGCTTGCGCACGGTGAAACAGAACTTCTTGAACTCTCCGGCGCTGCCACTGCGTTCGTAGATCGTCTTGAACAACCATCTGGCTTCTGCCTTGCCTGCGGCGCGGCGTGCCAGGCGGTAGAGAAAACGGCCGATGCCGGGATCGATCAAAAAGTAATCGGGGTGAACCGTCAGCACGTCCGGGTTCTTGCCTTCTGTGATCTCGCGGTACATCCAATCAGCTAGCTCGATCTCGATGTACTCCGGCCGCCCGGTTTCGCTCTTTACGATCTTGTAGCGGCTAATCAAGGCTTCACCCTCGGATACCGTCACCAGGCGGCCGTTCTTGGCCTTCTTCGTACGCTGCATGGCAACGTGCGTGGTGTTTAACCGAATGCAGGTTTCTACCAGGTCGTCTTTCTGCTTTCCGCCATCGGCTCGCCGGCAGAACTTGAGTACGTCCGCAACGTGTGGACGGAACACGCGGCCGGGCTTGTCTCCCTTCCCTTCCCGGTATCGGTTCATGGATTCGGTTAGATGGGAAACCGCCATCAGTACCAGGTCGTAATCCCACACACTGGCCATGCCGGCCGGCCCTGCGGAAACCTCTACGTGCCCGTCTGGAAGCTCGTAGCGGATCACCTCGCCAGCTCGTCGGTCACGCTTCGACAGACGGAAAACGGCCACGTCCATGATGCTGCGACTATCGCGGGTGCCCACGTCATAGAGCATCGGAACGAAAAAATCTGGTTGCTCGTCGCCCTTGGGCGGCTTCCTAATCGACGGCGCACCGGCTGCCGGCGGTTGCCGGGATTCTTTGCGGATTCGATCAGCGGCCGCTTGCCACGATTCACCGGGGCGTGCTTCTGCCTCGATGCGTTGCCGCTGGGCGGCCTGCGCGGCCTTCAACTTCTCCACCAGGTCATCACCCAGCGCCGCGCCGATTTGTACCGGGCCGGATGGTTTGCGACCGCTCACGCCGATTCCTCGGGCTTGGGGGTTCCAGTGCCATTGCAGGGCCGGCAGGCAACCCAGCCGCTTACGCCTGGCCAACCGCCCGTTCCTCCACACATGGGGCATTCCACGGCGTCGGTGCCTGGTTGTTCTTGATTTTCCATGCCGCCTCCTTTAGCCGCTAAAATTCATCTACTCATTTATTCATTTGCTCATTTACTCTGGTAGCTGCGCGATGTATTCAGATAGCAGCTCGGTAATGGTCTTGCCTTGGCGTACCGCGTACATCTTCAGCTTGGTGTGATCCTCCGCCGGCAACTGAAAGTTGACCCGCTTCATGGCTGGCGTGTCTGCCAGGCTGGCCAACGTTGCAGCCTTGCTGCTGCGTGCGCTCGGACGGCCGGCACTTAGCGTGTTTGTGCTTTTGCTCATTTTCTCTTTACCTCATTAACTCAAATGAGTTTTGATTTAATTTCAGCGGCCAGCGCCTGGACCTCGCGGGCAGCGTCGCCCTCGGGTTCTGATTCAAGAACGGTTGTGCCGGCGGCGGCAGTGCCTGGGTAGCTCACGCGCTGCGTGATACGGGACTCAAGAATGGGCAGCTCGTACCCGGCCAGCGCCTCGGCAACCTCACCGCCGATGCGCGTGCCTTTGATCGCCCGCGACACGACAAAGGCCGCTTGTAGCCTTCCATCCGTGACCTCAATGCGCTGCTTAACCAGCTCCACCAGGTCGGCGGTGGCCCATATGTCGTAAGGGCTTGGCTGCACCGGAATCAGCACGAAGTCGGCTGCCTTGATCGCGGACACAGCCAAGTCCGCCGCCTGGGGCGCTCCGTCGATCACTACGAAGTCGCGCCGGCCGATGGCCTTCACGTCGCGGTCAATCGTCGGGCGGTCGATGCCGACAACGGTTAGCGGTTGATCTTCCCGCACGGCCGCCCAATCGCGGGCACTGCCCTGGGGATCGGAATCGACTAACAGAACATCGGCCCCGGCGAGTTGCAGGGCGCGGGCTAGATGGGTTGCGATGGTCGTCTTGCCTGACCCGCCTTTCTGGTTAAGTACAGCGATAACCTTCATGCGTTCCCCTTGCGTATTTGTTTATTTACTCATCGCATCATATACGCAGCGACCGCATGACGCAAGCTGTTTTACTCAAATACACATCACCTTTTTAGACGGCGGCGCTCGGTTTCTTCAGCGGCCAAGCTGGCCGGCCAGGCCGCCAGCTTGGCATCAGACAAACCGGCCAGGATTTCATGCAGCCGCACGGTTGAGACGTGCGCGGGCGGCTCGAACACGTACCCGGCCGCGATCATCTCCGCCTCGATCTCTTCGGTAATGAAAAACGGTTCGTCCTGGCCGTCCTGGTGCGGTTTCATGCTTGTTCCTCTTGGCGTTCATTCTCGGCGGCCGCCAGGGCGTCGGCCTCGGTCAATGCGTCCTCACGGAAGGCACCGCGCCGCCTGGCCTCGGTGGGCGTCACTTCCTCGCTGCGCTCAAGTGCGCGGTACAGGGTCGAGCGATGCACGCCAAGCAGTGCAGCCGCCTCTTTCACGGTGCGGCCTTCCTGGTCGATCAGCTCGCGGGCGTGCGCGATCTGTGCCGGGGTGAGGGTAGGGCGGGGGCCAAACTTCACGCCTCGGGCCTTGGCGGCCTCGCGCCCGCTCCGGGTGCGGTCGATGATTAGGGAACGCTCGAACTCGGCAATGCCGGCGAACACGGTCAACACCATGCGGCCGGCCGGCGTGGTGGTGTCGGCCCACGGCTCTGCCAGGCTACGCAGGCCCGCGCCGGCCTCCTGGATGCGCTCGGCAATGTCCAGTAGGTCGCGGGTGCTGCGGGCCAGGCGGTCTAGCCTGGTCACTGTCACAACGTCGCCAGGGCGTAGGTGGTCAAGCATCCTGGCCAGCTCCGGGCGGTCGCGCCTGGTGCCGGTGATCTTCTCGGAAAACAGCTTGGTGCAGCCGGCCGCGTGCAGTTCGGCCCGTTGGTTGGTCAAGTCCTGGTCGTCGGTGCTGACGCGGGCATAGCCCAGCAGGCCAGCGGCGGCGCTCTTGTTCATGGCGTAATGTCTCCGGTTCTAGTCGCAAGTATTCTACTTTATGCGACTAAAACACGCGACAAGAAAACGCCAGGAAAAGGGCAGGGCGGCAGCCTGTCGCGTAACTTAGGACTTGTGCGACATGTCGTTTTCAGAAGACGGCTGCACTGAACGTCAGAAGCCGACTGCACTATAGCAGCGGAGGGGTTGGATCAAAGTACTTTGATCCCGAGGGGAACCCTGTGGTTGGCATGCACATACAAATGGACGAACGGATAAACCTTTTCACGCCCTTTTAAATATCCGATTATTCTAATAAACGCTCTTTTCTCTTAG
